# Intraspecific variation among Chinook Salmon populations indicates physiological adaptation to local environmental conditions

**DOI:** 10.1093/conphys/coad044

**Published:** 2023-06-20

**Authors:** Kenneth W Zillig, Alyssa M FitzGerald, Robert A Lusardi, Dennis E Cocherell, Nann A Fangue

**Affiliations:** Department of Wildlife, Fish and Conservation Biology, University of California, Davis, CA 95616, USA; Institute of Marine Sciences, University of California Santa Cruz, Santa Cruz, CA 95064, USA; Fisheries Ecology Division, Southwest Fisheries Science Center, National Marine Fisheries Service, National Oceanic and Atmospheric Administration, Santa Cruz, CA 95060, USA; Department of Wildlife, Fish and Conservation Biology, University of California, Davis, CA 95616, USA; Center for Watershed Sciences, University of California, Davis, CA 95616, USA; Department of Wildlife, Fish and Conservation Biology, University of California, Davis, CA 95616, USA; Department of Wildlife, Fish and Conservation Biology, University of California, Davis, CA 95616, USA

**Keywords:** Aerobic scope, coldwater fishes, CTmax, growth rate, local adaptation

## Abstract

Understanding interpopulation variation is important to predicting species responses to climate change. Recent research has revealed interpopulation variation among several species of Pacific salmonids; however, the environmental drivers of population differences remain elusive. We tested for local adaptation and countergradient variation by assessing interpopulation variation among six populations of fall-run Chinook Salmon from the western United States. Juvenile fish were reared at three temperatures (11, 16 and 20°C), and five physiological metrics were measured (routine and maximum metabolic rate, aerobic scope, growth rate and critical thermal maximum). We then tested associations between these physiological metrics and 15 environmental characteristics (e.g. rearing temperature, latitude, migration distance, etc.). Statistical associations between the five physiological metrics and 15 environmental characteristics supported our hypotheses of local adaptation. Notably, latitude was a poor predictor of population physiology. Instead, our results demonstrate that populations from warmer habitats exhibit higher thermal tolerance (i.e. critical thermal maxima), faster growth when warm acclimated and greater aerobic capacity at high temperatures. Additionally, populations with longer migrations exhibit higher metabolic capacity. However, overall metabolic capacity declined with warm acclimation, indicating that future climate change may reduce metabolic capacity, negatively affecting long-migrating populations. Linking physiological traits to environmental characteristics enables flexible, population-specific management of disparate populations in response to local conditions.

## Teaser

Chinook Salmon exist as multiple distinct populations across the landscape and exhibit population-specific physiological traits (upper thermal tolerance, metabolic capacity and growth rate) consistent with a hypothesis of local adaptation to environmental conditions. Differences in thermal performance among Chinook Salmon have important implications for conservation and management.

## Introduction

A core pursuit in ecology is the investigation of biological variation. Manifestations of variation between or within species can provide valuable insight into how organisms optimize fitness and maintain physiological homeostasis in response to environmental pressure (Piersma and Drent, 2003). Phenotypic variation among organisms is a product of genetic variation and environment-induced plasticity (West-Eberhard, 1989). In the wild, disentangling the drivers of phenotypic variation is complicated if the genetic drivers of trait performance covary with relevant environmental clines (Conover and Schultz, 1995). Studying variation among populations controls for shared species’ physiology and evolutionary history, permitting greater power in identifying selective pressures and prescribing phenotypic variation to genetic diversity or environmental drivers. Rapid environmental change is altering habitats globally (IPCC, 2022), and therefore, understanding environmental characteristics that drive variation among populations enables us to predict diverse responses to a shifting environment (Crozier and Hutchings, 2014).

Diverse, population-specific responses to environmental change challenge our capability to predict population response and promote effective species conservation (Gayeski *et al.,* 2018; Zillig *et al.,* 2021). Population-level phenotypic variation may stem from adaptation to local environmental characteristics (Lonsdale and Levinton, 1985; Via *et al.,* 1995; Ridgway, 2001; Fangue *et al.,* 2006; Eliason *et al.,* 2011; Nyboer *et al.,* 2020). For example, populations from warm range boundaries may perform better in warm environments than populations from cold range boundaries. Alternatively, phenotypic variation among populations may be small or absent in the wild but emerges when populations are reared under shared conditions. So-named countergradient variation exists when the evolutionary response along an environmental gradient counters the phenotypic effects of that gradient (e.g. latitude [Conover and Present, 1990], temperature [Levins, 1969] or abundance of predators [Arendt and Wilson, 1999]). For instance, high-latitude populations of Atlantic Silversides (*Menidia menidia*) grow faster than populations from low latitudes when experimentally reared at the same temperatures, a response that compensates for the shortened growing season experienced at higher latitudes (Conover and Present, 1990). Interpopulation variation may also manifest as reversible plastic changes, with populations acclimatizing to local conditions (Via *et al.,* 1995). Interpopulation variation in thermal performance, whether due to local adaptation, acclimation or countergradient evolution, can lead to trade-offs in physiological performance (Feder, 1978; 10.1126/science.1083073, 2003; Comte and Olden, 2017) and divergent outcomes as the environment warms. Local adaptation to warm temperatures at low latitudes may provide for resilience to future warming, whereas locally adapted, high-latitude, cold-temperature physiologies may be unable to acclimate. Alternatively, if populations exhibit countergradient variation, high-latitude populations may improve their performance relative to their low-latitude counterparts. Understanding the patterns of intraspecific variation allows for improved prediction of species’ responses to climate change (Scott and Poynter, 1991; Jeffree and Jeffree, 1996; Schulte *et al.,* 2011).

Pacific salmonids (*Oncorhynchus* spp.) are a commercially and culturally important clade of climate-vulnerable species that require a population-specific approach for effective conservation (Gayeski *et al.,* 2018; Zillig *et al.,* 2021). Most anadromous salmonid species migrate to specific natal streams as adults for spawning, reducing gene flow among populations (Quinn, 2018) and allowing for genetic drift and the development of population-specific traits maximizing fitness to local environmental conditions. Population traits may vary according to latitude (e.g. size [Quinn, 2018]), migration difficulty (e.g. aerobic scope [Eliason *et al.,* 2011]), migratory phenotype (Zillig *et al.,* 2023) or river entry timing or migration distance (e.g. percentage of body fat [Crossin *et al.,* 2004; Quinn, 2018]). Data suggesting local adaption have been identified across multiple species of salmonid (Fraser *et al.,* 2011) including Steelhead Trout (*O. mykiss,* [Garvin *et al.,* 2015; Chen *et al.,* 2018; Micheletti *et al.,* 2018]), Cutthroat Trout (*O. clarkii,* [Drinan *et al.,* 2012; Anlauf-Dunn *et al.,* 2022]) and Sockeye Salmon (*O. nerka,* [Eliason *et al.,* 2011; Chen *et al.,* 2013]). Meanwhile, there is evidence for countergradient variation among populations of Brown Trout (*Salvelinus trutta,* [Álvarez *et al.,* 2006]) and Arctic Char (*S. alpinus,* [Chavarie *et al.,* 2010; Sinnatamby *et al.,* 2015]). While variation among salmonid populations is observed, we have not fully resolved the drivers of intraspecific variation, and therefore cannot predict population-specific physiology or responses to climate change.

Anthropogenic modification of freshwater ecosystems and the global impacts of climate change endanger the persistence of Pacific salmonid populations (Martins *et al.,* 2011; Moyle *et al.,* 2017; Reid *et al.,* 2019). Drivers of species decline include impassable man-made barriers, habitat degradation, overexploitation, and flow modification (Dudgeon *et al.,* 2006; Reid *et al.,* 2019). Increasing river temperatures, hypoxia, persistent droughts, warm-adapted non-native species, and novel pathogens exacerbate risk to salmonids both regionally and globally (Moyle *et al.,* 2017; Reid *et al.,* 2019; Lehman *et al.,* 2020; Akbarzadeh *et al.,* 2021; Mauduit *et al.,* 2022). A 2007 survey of Pacific salmon populations in the western contiguous United States found that 29% of populations have been lost since Euro-American contact (Gustafson *et al.,* 2007). The most imperiled species is Chinook Salmon (*O. tshawytscha*) having lost 40% of historical populations (159 of 396) and the most imperiled population, the Sacramento River winter-run, exhibits thermal physiology suited to its unique life-history strategy, but which exacerbates the risk of anthropogenic change (Zillig *et al.,* 2023). Effective management of remaining salmonids necessitates understanding intraspecific variation and its associated environmental drivers.

**Figure 1:**
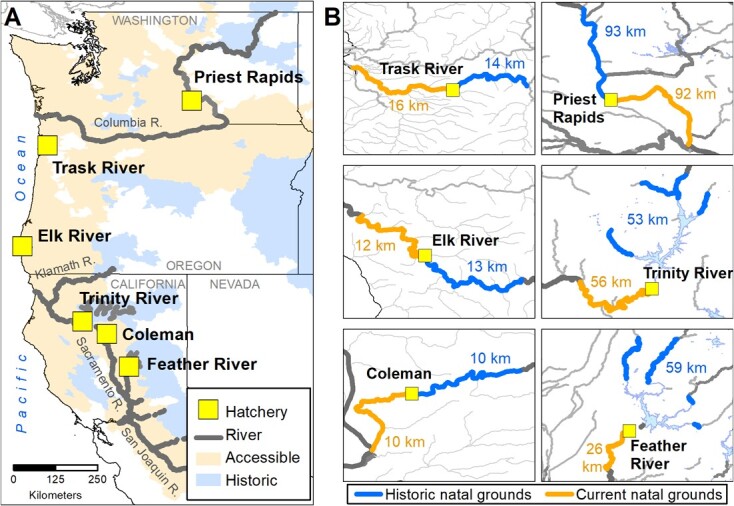
Map of studied populations. (A) Locations of the six hatchery populations in our study, shown against the Chinook Salmon accessible, current range, and their historically occupied range. Only major Chinook Salmon rivers are included for clarity. (B) The insets show the historic and current natal grounds surrounding each hatchery, and the respective number of river km analyzed for stream temperatures.

This study assessed patterns in interpopulation variation (local adaptation vs. countergradient variation) among six fall-run Chinook Salmon populations from Washington, Oregon and California (Fig. 1A, Table 1) by quantifying associations of physiological traits with population-specific characteristics of latitude, temperature and migratory challenges. We tested for relationships between physiological trait values and 15 environmental and thermal characteristics (e.g. latitude, outmigration route length, annual maximum temperature average temperature during rearing period) specific to each population’s historical rearing range upstream of impassable barriers, and to their current rearing range (i.e. below impassable barriers) (Fig. 1B, Table 2). Adult fall-run fish return to freshwater in autumn and spawn quickly upon arriving to their natal reaches. Fall-run embryos hatch in the late fall and early winter with juveniles typically rearing in freshwater for several months before outmigrating during their first spring. Among the studied populations, outmigration distances range from 22 to 630 km and rearing temperatures vary from 5.8 to 23.0°C depending on the population and month of year (FitzGerald *et al.,* 2020).

**Table 1:** Population and acclimation treatment metadata for six populations of Chinook Salmon

Population	State	ESU	Hatchery	Hatchery coord.	Elev. (m)	Migration distance (km)	Core rearing months	Rearing window range	Hatching date
Coleman	CA	California Central Valley Fall-run ESU	Coleman National Fish Hatchery	40.398°N, 122.145°W	123	441	Feb–Apr	Dec–Jun	08 Nov 2016
Elk River	OR	Southern Oregon and Northern California Coastal ESU	Elk River Hatchery	42.740°N, 124.403°W	35	22	Mar–Apr	Dec–Oct	14 Jan 2017
Feather River	CA	California Central Valley Fall-run ESU	Feather River Hatchery	39.519°N, 121.554°W	41	233	Feb–Apr	Dec–Jun	29 Nov 2018
Priest Rapids	WA	Upper Columbia Fall-run ESU	Priest Rapids Hatchery	46.630°N, 119.872°W	125	631	Mar–Apr	Feb–Aug	01 Feb 2018
Trask River	OR	Oregon Coastal ESU	Trask Hatchery	45.433°N, 123.726°W	17	28	Mar–Apr	Dec–Oct	14 Dec 2016
Trinity River	CA	Upper Klamath-Trinity Rivers Fall-run ESU	Trinity River Hatchery	40.727°N, 122.795°W	562	250	Feb–Mar	Nov–Jun	21 Jan 2019

**Table 2:** Environmental predictors used in models detecting associations between physiological traits and environmental parameters

**Environmental Predictor**	**Abbreviation**	**Description**
Latitude ^G^		Latitude of the hatchery for each population
Migration Distance ^R^	Mig.D	River length in kilometers from the hatchery to tidally influenced waters.
Migration Slope	Mig.S	The average slope of the river, calculated by dividing the distance from the hatchery to tidally influenced waters (km) by the elevation (m) on google earth.
Annual Mean Monthly Maximum stream temperature ^N^	CAMax (Current), HAMax (Historical)	Average of maximum monthly temperature for each river kilometer for reaches below (Current) or above (Historical) dams
Annual Mean Monthly Minimum stream temperature ^N^	CAMin (Current), HAMin (Historical)	Average of minimum monthly temperature for each river kilometer for reaches below (Current) or above (Historical) dams
Annual Temperature Range stream temperature ^N^	CARange (Current), HARange (Historical)	Average differences between the minimum and maximum monthly temperature for each river kilometer for reaches below (Current) or above (Historical) dams
Rearing Season Maximum Monthly Average stream temperature ^N,P^	CRMax (Current), HRMax (Historical)	Average maximum monthly temperature for each river kilometer for reaches below (Current) or above (Historical) dams, limited to the months of juvenile rearing.
Rearing Core Maximum Monthly Average stream temperature ^N,P^	CRCMax (Current), HRCMax (Historical)	Average maximum monthly temperature for each river kilometer for reaches below (Current) or above (Historical) dams, limited to the month of peak juvenile emergence and the subsequent month.
Rearing Season Average Monthly Average stream temperature ^N,P^	CRAve (Current), HRAve (Historical)	Average of monthly temperatures for each river kilometer for reaches below (Current) or above (Historical) dams, limited to the months of juvenile rearing.

Abbreviations are those referenced in the manuscript. G indicates data was gathered from google earth. N indicates data was extracted from the stream temperature model developed by Isaak *et al*. (2017) and augmented by FitzGerald *et al*. (2020). P indicates that temperature data was limited to the period of juvenile rearing based upon phenology data from FitzGerald *et al*., 2020. R indicates that data was sourced using the R package ‘dataRetrieval’. ‘Current’ indicates reaches below dams or hatcheries; ‘Historical’ indicates reaches upstream of dams or hatcheries (see text for full description). Population reach meta data, mean, and standard deviation values for the stream model for each population are in Supplementary Table S8 & S15.

Chinook Salmon juveniles were reared at three ecologically relevant temperatures (11, 16 and 20°C) and five physiological metrics were assessed: growth rate, critical thermal maximum (CT_max_), routine metabolic rate (RMR), maximum metabolic rate (MMR), and aerobic scope (AS). Growth rate is a temperature dependent, holistic physiological trait, which varies among populations (Sogard *et al.,* 2012; Bærum *et al.,* 2016) and is used by resource managers to assess habitat suitability (Myrick and Cech, 2001; Marine and Cech, 2004). Similarly, CT_max_ is a standardized physiological metric of acute thermal tolerance (Beitinger *et al.,* 2000). AS, the difference between an organism’s RMR and MMR, quantifies the aerobic metabolic capacity. In ectotherms, AS is temperature dependent, and evaluating AS along a temperature gradient reveals how a population may respond to changes in the thermal environment (Schulte, 2015). Past research has used these physiological metrics to assess patterns of local adaptation and countergradient variation in other telosts (Unwin, 1997; Fangue *et al.,* 2006; Eliason *et al.,* 2011; Chen *et al.,* 2015; Verhille *et al.,* 2016; Poletto *et al.,* 2017).

We hypothesized that juvenile Chinook Salmon would exhibit interpopulation variation associated with habitat characteristics (e.g. stream temperature, outmigration distance), although whether these patterns would implicate local adaptation, countergradient variation or plastic acclimatory responses as the driver of variation was unknown. Should populations be locally adapted, we predicted greater thermal performance (e.g. increased CT_max_, greater warm-acclimated aerobic capacity, etc.) from populations from warmer habitats (Eliason *et al.,* 2011; Chen *et al.,* 2015, 2018). If populations exhibit countergradient variation across latitude, then northern populations should exhibit accelerated growth relative to southern populations to compensate for the shortened growing season at higher latitudes (Conover and Present, 1990; Sinnatamby *et al.,* 2015). Finally, if populations differ due to reversible plastic changes, then we expect population differences to disappear under shared rearing conditions.

## Materials and Methods

### Experimental design

Fall-run Chinook Salmon from six hatchery populations (Fig. 1A, Table 1) were reared at three acclimation temperatures (11, 16, and 20°C), for a total of 18 treatment groups. These temperatures were chosen to be ecologically relevant to the conditions that a juvenile Chinook Salmon may encounter during rearing and outmigration (FitzGerald *et al.,* 2020). We evaluated two or three populations per year from 2017–2019. The Institutional Animal Care Committee of UC Davis (Protocol # 19928) approved this research.

### Environmental data

We tested for relationships between physiological performance and 15 environmental parameters including latitude, migration distance and slope, and twelve temperature metrics specific to the rearing ranges of each population (Table 2). Latitude and the elevation of the hatcheries were determined using google earth. Migration distance was calculated using the R package ‘dataRetrieval’, which accesses the National Water Information System to provide hydrological data (De Cicco *et al.,* 2022). Migration slope was calculated by dividing the elevation of a population’s hatchery by its migration distance. Stream temperature metrics were extracted from the predictions of a spatial stream network (SSN) model (Isaak *et al.,* 2017; FitzGerald *et al.,* 2020). In brief, the SSN model is a specialized regression model that accounts for the complex autocorrelation in streams caused by directed flow and network connectivity. The SSN model performed well (r^2^ = 0.928) and was used to predict mean monthly stream temperature for 465 775 river km in the western U.S. (FitzGerald *et al.,* 2020).

Here, we extracted mean monthly stream temperature (average of 2002–2011) for each river kilometer within a population’s current rearing distribution. Juvenile rearing habitat was defined for each population from observed spatial distributions of spawning and rearing (FitzGerald *et al.,* 2020). We calculated the annual maximum, minimum, and range of mean stream temperatures for each population. However, the vast majority of fall-run juveniles in the Central Valley outmigrate from the natal grounds a few weeks or months after emergence (Williams, 2006; FitzGerald *et al.,* 2020; Sturrock *et al.,* 2020). We therefore calculated the maximum and average stream temperatures experienced by each population during its rearing range (defined as the months when juveniles are observed rearing) and during core rearing (defined as the month of peak emergence and the subsequent month). Rearing phenology was population-specific (Table 1), based on a literature review of Chinook Salmon phenology (FitzGerald *et al.,* 2020).

The rearing grounds of these populations are truncated by an adjacent dam or hatchery structure. To approximate the historical pre-dam thermal regimes for each population, we modeled the same thermal metrics on the stream reaches upstream of the dam or hatchery (Fig. 1B, Table 2). Pre-dam historical rearing distribution data are relatively poor (FitzGerald *et al.,* 2020), so to define potential rearing habitat upstream of dams, we eliminated all upstream reaches that were inhabitable due to river slope, flow, natural barriers or intermittency (Bjornn and Reiser, 1991; Agrawal *et al.,* 2005; Isaak *et al.,* 2017). If multiple upstream tributaries had potential rearing habitat, we only considered major tributaries where rearing or spawning were documented (Yoshiyama *et al.,* 2001).

### Fish husbandry

The studied juvenile Chinook Salmon were from six fall-run populations (Table 1). Juveniles came from six hatcheries from five defined evolutionary significant units (ESU, Waples, 1995). Fish from the Priest Rapids population were received as eyed eggs via overnight mail and surface sterilized with iodophor upon arrival. Fish from the Coleman population were acquired as eggs and trucked to the Center for Aquatic Biology and Aquaculture at UC Davis (CABA). Eggs and hatched alevin were incubated at 9°C until exogenous feeding began. Fish from all other populations were acquired from their respective hatcheries when of transportable size (~1-2 g) and trucked to the CABA in a 765-L tank. Oxygen was supplied with aeration and water temperatures were reduced with bagged ice as needed during transit. At CABA rearing and treatment tanks were supplied with temperature-controlled, fresh water from a dedicated well that was aerated with air stones. Fry from all populations were reared at 11–13°C until distributed into their acclimation treatment tanks (two 400 L tanks per acclimation temperature, *n* = 55 to 105 per tank). Fish were exposed to natural photoperiods (38°55’N Latitude) and were fed to excess with 2–4 mm Sinking Salmon Feed (Skretting, USA) using automated belt feeders to deliver food continuously during daylight hours. Rations (4% of body mass per day) were updated biweekly to account for fish growth and tank density. Acclimation temperatures were achieved by increasing tank temperature by ~ 1.5°C per day. Once tanks achieved their specific acclimation temperature fish were acclimated for at least three weeks prior to any experimental data collecstion. Mean tank temperatures (± SEM) were 11.1 ± 0.03°C (*n* = 12), 16.0 ± 0.03°C (*n* = 12) and 19.8 ± 0.03°C (*n* = 12). Tank temperatures were maintained for the duration of the experiments (4–7 months).

### Growth

Growth measurements were conducted biweekly until CT_max_ and metabolic experiments began. CT_max_ and metabolic experiments necessitated size-selection and biased any further growth data. Fish started at 7.90 ± 0.20g in mass and 8.45 ± 0.07 cm in fork length and grew to 14.10 ± 0.47 g and 10.2 ± 0.13 cm (mean ± SEM, 18 treatments). Every two weeks an arbitrary sample of 30 fish from each treatment (*n* = 15 per tank, *n* = 2149 total measurements) were measured for mass (± 0.01 grams, Ohaus B3000D) and fork length (± 0.1 cm) and then returned to their rearing tank. The same experimenter netted and measured all fish. Fish were not individually marked and therefore growth rate was calculated at the treatment level. Time was defined as days since the first measurement point. Population specific growth rate data are contained in Table 3.

**Table 3:** Growth rate data for six population of Chinook Salmon at three acclimation temperatures

Hatchery & Acclimation Temperature (°C)		Initial Date	Final Date	Time (Days)	Mass (g)		Fork Length (cm)		Condition Factor		Growth Rate (g/day)	Acclimation Capacity (∆g/day)
					Initial	Final	Initial	Final	Initial	Final		
	11	4/17/17	5/16/17	29	7.45 ± 0.50	11.41 ± 2.69	8.3 ± 0.8	9.7 ± 0.8	1.26 ± 0.09	1.21 ± 0.06	0.16 ± 0.021	
Coleman	16	4/17/17	5/16/17	29	8.42 ± 0.09	15.69 ± 3.24	8.8 ± 0.8	10.8 ± 0.7	1.23 ± 0.11	1.22 ± 0.07	0.23 ± 0.026	0.11 ± 0.028
	20	4/17/17	5/16/17	29	8.34 ± 0.02	17.29 ± 3.56	8.7 ± 0.6	11.1 ± 0.7	1.27 ± 0.08	1.26 ± 0.08	0.27 ± 0.026	
	11	7/25/17	9/4/17	41	6.81 ± 1.93	14.81 ± 4.85	8.2 ± 0.8	10.4 ± 1.1	1.20 ± 0.14	1.28 ± 0.07	0.17 ± 0.023	
Elk River	16	7/13/17	8/8/17	26	8.30 ± 2.48	13.79 ± 4.36	8.6 ± 0.8	10.1 ± 1.0	1.27 ± 0.08	1.29 ± 0.08	0.22 ± 0.039	0.04 ± 0.032
	20	6/28/17	8/8/17	41	6.99 ± 1.36	15.71 ± 4.96	8.0 ± 0.5	10.5 ± 1.0	1.33 ± 0.07	1.31 ± 0.07	0.21 ± 0.025	
	11	5/20/19	7/1/19	42	8.23 ± 2.92	16.01 ± 4.88	8.6 ± 1.0	10.7 ± 1.0	1.24 ± 0.08	1.26 ± 0.09	0.17 ± 0.023	
Feather River	16	5/6/19	6/3/19	28	8.83 ± 3.88	13.17 ± 5.90	8.5 ± 1.4	9.9 ± 1.6	1.31 ± 0.09	1.26 ± 0.10	0.17 ± 0.036	0.06 ± 0.042
	20	5/6/19	6/3/19	28	8.44 ± 3.43	14.90 ± 5.39	8.4 ± 1.1	10.3 ± 1.3	1.32 ± 0.11	1.29 ± 0.07	0.24 ± 0.037	
	11	7/12/18	8/23/18	42	7.34 ± 3.46	12.77 ± 5.85	8.4 ± 1.3	10.0 ± 1.5	1.13 ± 0.06	1.18 ± 0.05	0.13 ± 0.022	
Priest Rapids	16	6/27/18	7/26/18	29	8.02 ± 2.92	14.50 ± 7.04	8.5 ± 1.2	10.3 ± 1.6	1.27 ± 0.33	1.23 ± 0.07	0.22 ± 0.035	0.08 ± 0.032
	20	7/12/18	8/23/18	42	7.43 ± 3.44	15.62 ± 7.70	8.4 ± 1.6	10.4 ± 1.9	1.19 ± 0.05	1.26 ± 0.06	0.21 ± 0.024	
	11	8/8/17	9/19/17	42	7.80 ± 3.00	14.64 ± 5.40	8.5 ± 1.0	10.6 ± 1.2	1.20 ± 0.08	1.19 ± 0.08	0.16 ± 0.023	
Trask River	16	7/25/17	8/31/17	37	7.11 ± 1.95	14.77 ± 5.32	8.2 ± 0.7	10.3 ± 1.0	1.24 ± 0.08	1.29 ± 0.11	0.22 ± 0.026	0.02 ± 0.033
	20	8/8/17	9/19/17	42	8.62 ± 3.34	15.81 ± 6.01	8.7 ± 1.0	10.7 ± 1.2	1.22 ± 0.13	1.24 ± 0.09	0.18 ± 0.026	
	11	8/12/19	9/9/19	28	7.94 ± 3.93	9.50 ± 5.35	8.6 ± 1.4	9.0 ± 1.8	1.17 ± 0.05	1.15 ± 0.06	0.06 ± 0.032	
Trinity River	16	7/17/19	9/9/19	54	7.19 ± 4.07	11.29 ± 9.33	8.1 ± 1.5	9.1 ± 2.4	1.21 ± 0.07	1.2 ± 0.08	0.07 ± 0.018	0.06 ± 0.036
	20	7/17/19	9/9/19	54	6.21 ± 3.57	12.18 ± 7.09	7.9 ± 1.4	9.6 ± 1.8	1.18 ± 0.11	1.22 ± 0.05	0.12 ± 0.018	

Mass, fork length and Fulton’s condition factor are all reported as means and standard deviations of the observed data at the beginning (Initial) and end (Final) of the measurement window. Growth rate is the modeled growth rate reported as the mean and standard deviation of 28 000 draws from the posterior distribution of lowest WAIC growth model. Acclimation capacity is the mean and standard deviation of the contrast distribution of the 11 and 20°C acclimation groups.

### Critical thermal maximum

CT_max_ values were quantified according to established methods (Becker and Genoway, 1979). Six 4 L Pyrex beakers were placed in a fiberglass bath tray (1 m x 2 m x.2 m) and individually aerated with an air stone. The volume of water in each individual beaker (approx. 2.5 L) was calibrated to ensure even heating across all CT_max_ beakers (0.33°C min^−1^). Two pumps (PM700, Danner, USA) were used to circulate water: one pump recirculated water across three heaters (Process Technology S4229/P11), while the other distributed heated water through the CT_max_ bath via a distribution manifold. Experiments began with water temperature set at the fish’s acclimation temperatures (11, 16 or 20°C).

Fish were arbitrarily selected from treatment tanks (Mass: 23.03 ± 4.87 g, Fork length: 12.4 ± 0.8 cm, mean ± SD, *n* = 18 to 25 per treatment) and transferred to separate tanks for fasting. To ensure fish were in a similar postprandial state, fish reared at 20°C and 16°C were fasted for 24 hours and 11°C fish were fasted for 48 hours to account for their slower metabolic rate. After fasting, fish were individually netted and transferred into individual beakers within the CT_max_ heat bath. Fish were given 30 minutes to acclimate to their CT_max_ beaker after which the CT_max_ trial began.

During the CT_max_ trial, the temperature of each beaker was taken every 5 minutes using a thermocouple (Omega HH81A) routinely calibrated to a standardized thermometer. Fish were observed continually for signs of distress and loss of equilibrium. The CT_max_ trial endpoint was loss of equilibrium, at which point the temperature of the CT_max_ beaker was recorded (Beitinger *et al.,* 2000; Fangue *et al.,* 2006). Fish were then removed to a recovery bath at their acclimation temperature. Fish that did not fully recover within 24-hours were not included in analysis (6% of individuals). Fish were then euthanized in a buffered solution of MS-222 (0.5 g L^−1^) and then weighed (wet mass) and measured (fork length).

### Metabolic experiments

#### Respirometry

Fish (Mass: 23.92 ± 4.25 g, Fork Length: 12.6 ± 0.7 cm, mean ± SD, *n* = 32–46 per treatment) underwent metabolic trials in one of four, 5 L automated swim tunnel respirometers (Loligo, Denmark). Description of the swim tunnel system can be found in (Zillig *et al.,* 2023). Swim tunnels and associated sump systems were cleaned and sanitized with bleach weekly to reduce potential for bacterial growth. Fish from each population x acclimation temperature treatment group were acutely tested at a range of temperatures (8–26°C), each fish was only tested once (Supplementary Table S1).

Dissolved oxygen saturation within the swim tunnels was measured using fiber-optic dipping probes (Loligo OX11250), which continuously recorded data via AutoResp™ software (version 2.3.0). Oxygen probes were calibrated weekly using a two-point, temperature-paired calibration method using deionized water that was either aerated using a bubbler or deoxygenated with sodium sulfite. Water velocity of the swim tunnels was quantified and calibrated using a flowmeter (Hontzcsh, Germany), regulated using a variable frequency drive controller (models 4x and 12 K; SEW Eurodrive, USA) and controlled (precision < 1 cm s^−1^) via the Autoresp™ program and a DAQ-M data acquisition device (Loligo, Denmark). Swim tunnels were surrounded by shade cloth to reduce disturbance of the fish and remotely monitored by infrared cameras (QSC1352W; Q-see, China).

Oxygen consumption rates for both routine and maximal metabolic rates were captured using intermittent respirometry (Brett, 1964). A flush pump (Eheim 1048A, Germany) for each tunnel pumped aerated fresh water through the swim chamber and was automatically controlled via the AutoResp™ software. Computer-controlled sealing of the tunnel enabled the measurement of oxygen consumption attributable to the fish. Oxygen saturation levels were not allowed to drop below 80% and were restored quickly via the flush pump. Oxygen saturation data from AutoResp™ was transformed to oxygen concentration ([*O_2_*]: mgO_2_L^−1^):

Equation 1 \begin{align*} \left[{O}_2\right]=\frac{\%{O}_2 Sat}{100}\times \alpha \left({O}_2\right)\times BP \end{align*}

*%O_2_Sat* is the oxygen saturation percentage reported from AutoResp™; *α(O_2_)* is the coefficient of temperature-corrected oxygen solubility (mgO_2_ L^−1^ mmHg^−1^); and *BP* is the barometric pressure (mmHg). Oxygen concentration (mgO_2_ L^−1^) was measured every second and regressed over time; the coefficient of this relationship (*R*; mgO_2_ L^−1^ s^−1^) was then converted to metabolic rate (*MO_2_*; mgO_2_ kg^−1^ min^−1^, Equation 2).

Equation 2 \begin{equation*} M{O}_2=R\times V\times{M}^{-1}\times \mathrm{r} \end{equation*}

*V* is the volume of the closed respirometer (L); *M* is the mass of the fish (kg) and r (*60*s min^−1^) transforms the rate from per second to per minute. An allometric scaling exponent was not incorporated due to similarity in fish sizes (Poletto *et al.,* 2017). Collected oxygen concentration data was visually inspected for linearity. Median R^2^ for regressed RMR and MMR data were 0.980 and 0.995 respectively.

#### Routine metabolic rate (RMR)

Prior to RMR trials fish were fasted to ensure a post-prandial state using the same durations implemented during the CT_max_ trials. Afterwards, fish were transferred into a respirometer and provided a 30-minute acclimation period at their acclimation temperature (11, 16 or 20°C). The temperature was then adjusted (2°C h^−1^) from the acclimation temperature to one of 10–12 test temperatures (8, 10, …, 22, 24, 25, 26°C). The sequence of temperatures was arbitrary and sought to balance trials across the temperature range. Automated intermittent flow respirometry began 30 minutes after the test temperature was achieved and continued overnight. Measurement periods ranged between 900 to 1800 s in duration, flush periods were 180–300 s. Measurement and flush periods varied in length to accommodate for fish mass and test temperature, ensuring oxygen saturation was kept high (>80%) throughout the trial. A circulation pump (DC30A-1230, Shenzhen Zhongke, China) mixed water within the tunnel without disturbing the fish. RMR was calculated by averaging the three lowest MO_2_ values (Poletto *et al.,* 2017). Fish were monitored for overnight activity using continuous video recording and fish that exhibited swimming during the lowest RMR periods (*n* = 7) were discarded from the analysis. RMR experiments (*n* = 710) began between 13:00 and 17:00 and were concluded at 08:00 ± 40 min. We elected to report our values as RMRs instead of standard metabolic rates as our trials were short and fish were capable of some movement within the respirometer (Chabot *et al.,* 2016).

#### Maximum metabolic rate (MMR)

Immediately following RMR measurements, we implemented a modified critical swimming velocity protocol to elicit MMR from each fish (Poletto *et al.,* 2017). Tunnel velocity was increased from 0 to 30 cm s^−1^ over a ~ 2 min period and held for 20 min. For each subsequent 20-minute measurement window, tunnel velocity was increased 10% up to a maximum of 6 cm s^−1^ per step. Swimming metabolism was measured by sealing the tunnel for approximately 16 minutes of each measurement window. Tunnel oxygen levels were not allowed to drop below 80%. If a fish impinged upon the back screen (>2/3 of body in contact with screen) the tunnel velocity was stopped for one minute and subsequently returned to the original speed over the following two minutes. Exhaustion was defined as two impingements within the same velocity step. At this point the impeller was turned off and the tunnel was unsealed. The highest metabolic rate measured over a minimum of 5 min during swimming activity was taken as the MMR. AS was calculated as the difference between an individual’s RMR and MMR.

Post-experiment fish were placed into a recovery tank. After a 24-hour recovery period fish were euthanized in a buffered solution of MS-222 (0.5 g L^−1^). Measurements for mass (g), fork length (cm) and total length (cm) were taken, and Fulton’s condition factor was calculated (Froese, 2006). In seeking evidence of metabolic collapse at near-critical temperatures, some metabolic trials were conducted at temperatures exceeding the tolerance of the fish. These mortality events represent potential lethal upper limits for sub-acute thermal persistence (Supplementary Table S1). Data from fish that did not survive the trial or recovery were not included in analysis.

### Statistical analyses

We developed separate generalized linear mixed models (GLMMs) for each of the five physiological traits (CT_max_, Growth Rate, RMR, MMR and AS) to estimate mean treatment responses. All models assumed a Gaussian distribution for the response variable. All models included population and acclimation temperature as interacting categorical fixed predictors. Additional predictor variables and random effects were included depending on the response variable and model fit (see below). Stepwise model selection was used to identify the model with the lowest widely applicable information criteria (WAIC) to avoid overfitting (Supplementary Table S2-4). Models were visually checked for fit with the packages *ggplot2* (Wickham, 2016) and *tidybayes* (Kay, 2020). Relationships among current and historical rearing ranges and temperature data were assessed using simple linear models. All statistical analyses were conducted in R (version 4.0.2) using the package *brms* (Bürkner, 2017, 2018) to construct Bayesian GLMMs with weakly regulating priors.

**Table 4:** Treatment measurements for critical thermal maximum (CT_max_)

Hatchery and acclimation temperature (°C)		Modeled mean CTM (°C)	Observed CTM (°C)	Mass (g)	Fork length (cm)	Fulton’s condition factor	Count	Acclimation capacity (°C)
	11	28.1 ± 0.14	27.9 ± 0.39	17.21 ± 5.15	11.5 ± 1.1	1.11 ± 0.07	22	
Coleman	16	29.3 ± 0.15	29.3 ± 0.37	22.93 ± 3.37	12.4 ± 0.7	1.19 ± 0.06	20	1.9 ± 0.17
	20	30.0 ± 0.15	30.0 ± 0.40	23.26 ± 3.76	12.3 ± 0.7	1.24 ± 0.07	20	
	11	27.9 ± 0.15	28.0 ± 0.45	26.44 ± 3.	13.0 ± 0.5	1.19 ± 0.05	21	
Elk River	16	28.5 ± 0.14	28.5 ± 0.83	24.91 ± 2.9	12.7 ± 0.5	1.21 ± 0.09	20	1.5 ± 0.21
	20	29.5 ± 0.15	29.1 ± 0.91	26.90 ± 3.0	12.7 ± 0.4	1.31 ± 0.07	19	
	11	27.8 ± 0.13	27.8 ± 0.40	25.26 ± 2.4	13.1 ± 0.4	1.13 ± 0.04	21	
Feather River	16	29.0 ± 0.14	29.0 ± 0.53	22.03 ± 2.3	12.3 ± 0.4	1.18 ± 0.08	23	1.1 ± 0.20
	20	28.9 ± 0.15	28.7 ± 0.81	23.62 ± 2.9	12.2 ± 0.5	1.30 ± 0.11	22	
	11	27.9 ± 0.14	27.9 ± 0.46	18.84 ± 1.4	12.0 ± 0.3	1.09 ± 0.05	23	
Priest Rapids	16	28.9 ± 0.18	28.9 ± 0.70	20.67 ± 1.4	12.2 ± 0.3	1.13 ± 0.05	20	1.8 ± 0.19
	20	29.7 ± 0.16	29.6 ± 0.67	23.08 ± 3.34	12.5 ± 0.4	1.19 ± 0.11	20	
	11	28.0 ± 0.20	28.1 ± 0.43	25.69 ± 2.96	13.2 ± 0.5	1.12 ± 0.05	25	
Trask River	16	28.6 ± 0.17	28.4 ± 0.73	27.56 ± 4.2	13.2 ± 0.5	1.20 ± 0.07	20	2.1 ± 0.19
	20	30.1 ± 0.18	30.0 ± 0.77	26.08 ± 2.	12.8 ± 0.4	1.25 ± 0.09	18	
	11	28.4 ± 0.15	28.5 ± 0.18	18.92 ± 5.43	11.8 ± 1.1	1.13 ± 0.04	21	
Trinity River	16	28.9 ± 0.15	29.2 ± 0.66	18.59 ± 7.47	11.6 ± 1.5	1.14 ± 0.06	21	0.7 ± 0.19
	20	29.2 ± 0.14	29.1 ± 0.97	23.79 ± 5.84	12.4 ± 0.8	1.23 ± 0.09	21	

Modeled data is the mean and standard deviation of 14 000 draws from the posterior distribution. The remaining columns are the respective means and standard deviations of the specific fish trialed for CTmax.

Each physiological model was different to maximize fit. The final growth rate model incorporated mass as a linear function of time with an additional fixed effect for the starting mass of each treatment group (Supplementary Table S2). A random effect for rearing tank was tested but was not included in the lowest WAIC model. The final CT_max_ model (Supplementary Table S3) additionally included fixed effects for fish mass and age (days post hatch). The relationship between RMR and test temperature was fit to an exponential curve by log-transforming the RMR values (Supplementary Table S4). The final model included non-interacting fixed effects for swim-tunnel and fish age. The final MMR model (Supplementary Table S4) was fit to the log-transformation of test temperature with a fixed effect for swim-tunnel, Fulton’s condition factor, and fish age. The final AS model was defined by a second order polynomial function of test temperature and an additional fixed effect for Fulton’s condition factor (Supplementary Table S4). Mass, condition factor, test temperature and all response variables were centered and scaled to standard deviations (Z-scores). The predictor variables for time and fish age (days post hatch) were standardized to range from 0 to 1.

Using the lowest WAIC model for each physiological trait, means and standard deviations for each treatment group were calculated using the package *emmeans* (Lenth, 2020). The model posterior distributions were used to calculate pairwise differences in mean estimates of CT_max_ and growth rate between treatment groups. For each contrast, strong significance was assigned if 94.5% of the posterior distribution was above or below 0, and weak significance was assigned if 85% of the posterior distribution was above or below 0. Acclimation capacity, the difference in mean trait estimates between 11 and 20°C acclimated fish, were likewise quantified for each population using the posterior distributions of the final CT_max_ and growth rate models.

Values for the thermal optimum (T_opt_: the temperature at which AS is maximized) for each treatment group were calculated using 500 simulated datasets randomly sampled from the posterior distributions of the AS model. T_opt_ was calculated by fitting a quadratic equation to each simulated AS sample and then calculating the root of the first derivative, allowing for estimation of the mean and standard deviation of T_opt_ for each population.

We assessed the effect of 15 environmental predictor variables (Table 2) on each of the five physiological traits using GLMMs. For each physiological trait, we first developed models (Supplementary Table S5, S6, S7), that included latitude, a widely used bio-geographic predictor, and additional fixed effects (fish age, mass, body length etc.) and a random effect for source population. We identified the lowest-WAIC model and then constructed 14 additional models corresponding to the 14 remaining environmental predictors, replacing latitude with a given environmental predictor of interest. Due to high correlation among some environmental predictors (Supplementary Fig. S1), each model only contained a single environmental predictor variable. For example, the lowest-WAIC model of the association between latitude and CT_max_ included fixed effects for latitude, acclimation temperature, fish mass and fish age, and random effects for CT_max_ test chamber and hatchery (Supplementary Table S3). This model was then replicated 14 additional times, replacing latitude with a different environmental predictor. This process was repeated for all five physiological metrics. The resulting 75 models (15 models per five physiological traits) were then used to assess the association of environmental predictors with physiological traits for fish reared at each acclimation temperature (11, 16 or 20°C). For the three metabolic traits (RMR, MMR and AS), we additionally included an interaction of each environmental predictor variable with the test temperature of the metabolic trial (8–25°C), as well as the fixed effect for acclimation temperature. This allowed us to coarsely assess the association between a given environmental predictor and metabolic trait across a thermal gradient. For each of the three metabolic traits (RMR, MMR and AS) we report the effect of each predictor at three test temperatures (11, 16 and 20°C) per acclimation group (9 associations per environmental predictor per metabolic trait) for a total of 495 associations. For each association we determined directionality of effect (positive or negative) and attributed strong significance, weak significance, or no significance (as defined above).

## Results

### Environmental characteristics

When comparing historical (i.e. upstream of impassable barriers) and current (i.e. below barriers) river temperature for all six of our populations some consistent patterns emerged. In general, temperature metrics were higher within current rearing habitats than historical habitats (p < 0.001) (Supplementary Table S8). For example, the average temperature during juvenile rearing across populations increased from 7.5°C within historical habitats (HRAve) to 9.2°C within current habitats (CRAve). Among our environmental traits, temperature metrics were typically highly correlated (Correlation > 0.5). For example, streams exhibiting warmer maximum rearing temperatures were likely to have warmer annual maximum temperatures (e.g. CRMax vs. CAMax, corr. 0.9; Supplementary Fig. S1).

### Growth rate

**Figure 2:**
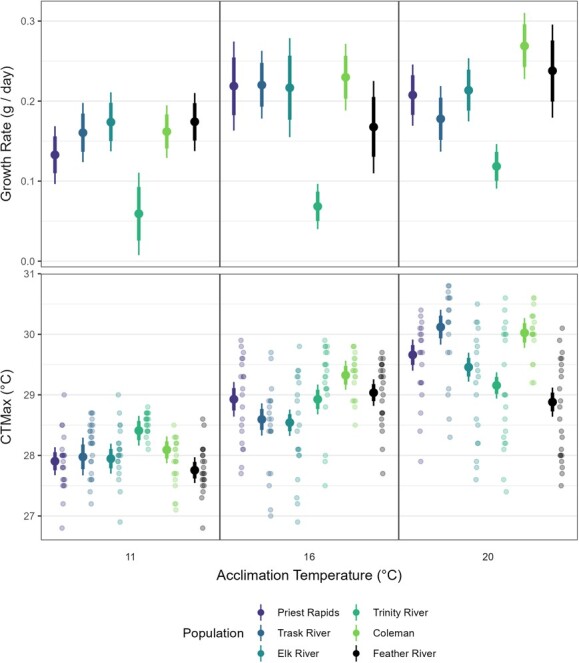
CT_max_ and growth rates of six populations of fall-run Chinook Salmon acclimated to three temperatures**.** (A) Modeled growth rates (g/day). (B) Observed (jittered individual points) and modeled CT_max_ values (°C). Populations are ordered by latitude (North to South). Mean model estimate is represented by the offset large point, while the 70% (thick) and 89% (narrow) credible intervals are represented by the whiskers.

Across all populations, growth rates were slowest in fish acclimated to 11°C and typically increased with acclimation to 16°C (Fig. 2A). The three southernmost populations (Coleman, Feather River, and Trinity River) exhibited increased growth rates when acclimated to 20°C, whereas the Elk River, Trask River and Priest Rapids populations exhibited non-significant declines in growth rate when acclimated to 20°C (Table 3). The Coleman population acclimated to 20°C had the fastest growth rate, although this value was not statistically different from the comparably acclimated Feather River population. See supplementary materials for pairwise comparisons of growth rate among treatments (Supplementary Fig. S2).

The slowest growth rates were exhibited by the Trinity River population, which grew significantly slower (< 5.5% posterior overlap with other treatment estimates) than any other comparably acclimated population. Despite overall slow growth, the Trinity River population did show an increased growth rate with acclimation temperature. Due to the slow growth of the Trinity River population, we ran all subsequent statistical analyses and models both including (Supplementary Table S9) and excluding the data from Trinity River (Supplementary Table S10) as this population may be demonstrating an alternate life-history strategy (see Discussion), thereby reducing its comparability to the other populations.

**Figure 3:**
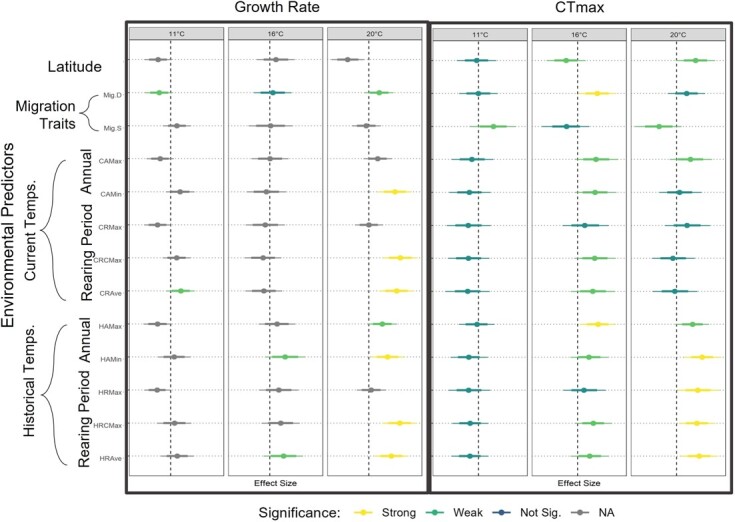
Associations of 15 environmental predictors with (A) growth and (B) critical thermal maximum (CT_max_). Acclimation temperatures are each assigned a vertical panel. Mean model estimate is represented by the offset large point, while the 70% (thick) and 89% (narrow) credible intervals are represented by the whiskers. Strong significance was assigned if ≥94.5% of the posterior distribution of the association was above or below 0. Weak significance was assigned if ≥85% of the posterior distribution was above or below 0. For associations between environmental predictors and growth, only estimates that were consistent to the inclusion or exclusion of Trinity hatchery were considered for significance. If a trait was not robust to the inclusion of Trinity hatchery data, it is colored gray.

Environmental predictors of growth rate were assessed for each acclimation temperature (Fig. 3A). Growth rate of fish acclimated to 11°C or 16°C did not exhibit any significant associations with thermal predictors. However, when populations were acclimated at 20°C, growth rate was positively associated with several current and historical stream temperature metrics (CRCMax, HRCMax, CCRAve, HCRAve, CAMin, HAMin). Migration distance was found to have weakly significant associations with growth rate, with a negative association at 11°C acclimation, no significant association at 16°C, and a positive association at 20°C.

### Critical thermal maximum

Acclimation temperature was a significant predictor of CT_max_. All populations exhibited significantly greater CT_max_ when acclimated to 16 vs. 11°C, and all populations excluding the Feather River population exhibited significantly greater CT_max_ when acclimated to 20 vs. 16°C (Fig. 2B).

The highest modeled CT_max_ values belonged to the Coleman and Trask River populations acclimated to 20°C (see Supplementary Fig. S3 for pairwise comparisons of CT_max_ among treatments). Within each population standard deviations of the observed CT_max_ values increased with acclimation temperature, with five of six populations exhibiting the greatest variation when acclimated to 20°C (Table 4).

The association of 15 environmental predictors with CT_max_ were assessed at each acclimation temperature (Fig. 3B). We did not find strong significant relationships between CT_max_ and any predictor variable among fish acclimated to 11°C. Fish acclimated to 16°C had strong significant positive associations with HAMax and Mig.D. There were weakly-significant but positive associations with several current and historical stream temperatures on the juvenile rearing grounds (CAMax, CAMin, CRCMax, CCRAve, HRCMax, HCRAve and HRMax). CT_max_ for fish acclimated to 20°C had strong significant positive associations with HAMin and historical estimates of maximum and average stream temperatures during juvenile rearing (HRAve, HRMax, HCRMax). There were weakly-significant but positive associations with latitude, and HAMax and CAMax. Historical estimates of stream temperature were more likely to be significantly associated with CT_max_ values than current estimates. For specific associations see Supplementary Table S11.

### Routine metabolic rate (RMR)

**Figure 4:**
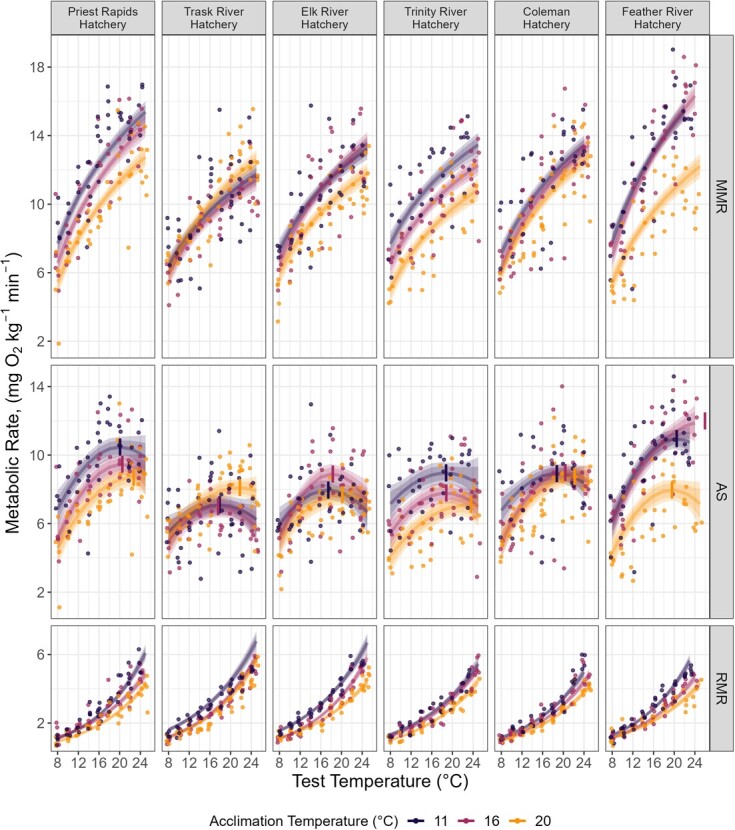
Metabolic rates for six populations of fall-run Chinook Salmon reared at three acclimation temperatures. An individual fish was tested at only one acute test temperature and elicited a single RMR and MMR, from which an AS could be calculated. Colors represent acclimation temperature groups. Point reference observed data and are jittered for visibility, while lines are the trait estimates derived from the lowest-WAIC model. Shaded regions represent the 70% (dark) and 89% (light) credible interval. Calculated thermal optima (T_opt_) are indicated by the vertical segments on the AS plots. Populations are organized from North (left) to South (right).

RMR increased with test temperature and was modeled using an exponential function of test temperature (Supplementary Table S4). In all populations, acclimating fish to warmer water temperatures reduced RMR rates across the range of test temperatures (Fig. 4). Acclimation to 20°C reduced the overall RMR of a given population to between 80.00% (Coleman) and 68.88% (Elk River) of the population’s RMR elicited when acclimated to 11°C (Table 5).

### Maximum metabolic rate (MMR)

MMR increased with test temperature and was best fit when modeled as a function of the base 2 logarithm of test temperature (Supplementary Table S4). Acclimation to warmer temperatures reduced overall MMR capacity in all populations except Trask River, which maintained similar MMR values across acclimation temperatures (Fig. 4, Table 5). Among the remaining five populations the negative effect of warm acclimation temperature varied. Coleman and Elk River populations exhibited a small effect of acclimation temperature, while the Priest Rapids, Feather River and Trinity populations exhibited more pronounced declines among fish reared at 20°C.

### Aerobic scope

The relationship of AS across temperature adhered to a typical thermal performance curve and was represented as a second order polynomial function of test temperature (Fig. 4, Supplementary Table S4). The thermal optima (T_opt_) of AS increased with acclimation temperature and ranged between 17.45 ± 0.41°C (Elk River population acclimated to 11°C) to 23.59 ± 2.50°C (Trinity River population acclimated to 20°C). In five of the six populations T_opt_ increased between fish acclimated to 11°C and those acclimated to 20°C (Table 5). The Feather River population was distinct in two regards, it was the only population to demonstrate a decline (−0.91°C) in T_opt_, although this decrease was not significant. Additionally. the AS curve for the Feather River population acclimated to 16°C was monotonic over the measured temperature range (8–25°C) and therefore the calculated T_opt_ (25.95 ± 2.22°C) exceeded the temperature at which we were able to test fish (Supplementary Table S1).

**Table 5:** Summary metabolic data for six populations of fall-run Chinook Salmon

Hatchery and acclimation temperature (°C)		Fish swum (n)	Mort. (n)	Max test Temp. (°C)	Mass (g)	Fork length (cm)	Fulton’s cond. factor	RMR		MMR	AS			
								Q_10_	% of 11 °C	% of 11 °C	AS at T_OPT_ (mgO_2_ kg^−1^ hr^−1^)	T_OPT_ (°C)	% of 11 °C	T_OPT_ ∆ (°C)
	11	32	7	24	22.18 ± 4.02	12.5 ± 0.7	1.13 ± 0.07	2.50 ± 0.03	-	-	8.92 ± 0.09	18.75 ± 0.64	-	
Coleman	16	42	3	25	23.72 ± 3.25	12.7 ± 0.5	1.16 ± 0.05	2.48 ± 0.02	89.06 ± 4.28	95.42 ± 4.89	8.98 ± 0.08	20.38 ± 0.48	94.15 ± 12	3.44 ± 1.08
	20	45	5	25	24.72 ± 4.01	12.6 ± 0.6	1.21 ± 0.06	2.34 ± 0.02	80.00 ± 4.78	90.86 ± 4.70	8.76 ± 0.09	22.19 ± 0.89	91.64 ± 11.82	
	11	39	5	24	26.72 ± 3.66	13.1 ± 0.5	1.19 ± 0.05	2.36 ± 0.03	-	-	7.96 ± 0.09	17.45 ± 0.41	-	
Elk River	16	39	2	24	23.80 ± 2.95	12.5 ± 0.5	1.20 ± 0.07	2.67 ± 0.03	79.44 ± 6.75	98.86 ± 5.86	8.89 ± 0.10	18.33 ± 0.28	107.05 ± 13	2.69 ± 0.72
	20	44	7	25	25.32 ± 3.03	12.5 ± 0.5	1.29 ± 0.10	2.23 ± 0.02	68.88 ± 4.01	87.01 ± 4.72	7.69 ± 0.09	20.14 ± 0.57	96.68 ± 12.45	
	11	39	4	23	25.36 ± 2.57	13.0 ± 0.4	1.14 ± 0.05	2.67 ± 0.03	-	-	10.96 ± 0.10	20.48 ± 0.69	-	
Feather River	16	35	5	24	24.09 ± 2.59	12.7 ± 0.4	1.17 ± 0.07	2.41 ± 0.03	81.99 ± 5.88	96.90 ± 5.58	11.99 ± 0.35	25.95 ± 2.22	100.94 ± 8.88	−0.91 ± 0.85
	20	38	12	25	26.08 ± 4.26	12.6 ± 0.5	1.30 ± 0.12	2.20 ± 0.03	75.74 ± 8.28	72.72 ± 4.05	7.97 ± 0.10	19.57 ± 0.48	72.44 ± 6.56	
	11	41	2	24	20.60 ± 3.96	12.4 ± 0.7	1.07 ± 0.04	2.74 ± 0.03	-	-	10.47 ± 0.10	20.07 ± 0.58	-	
Priest Rapids	16	40	5	24	22.74 ± 3.46	12.6 ± 0.5	1.13 ± 0.07	2.32 ± 0.03	91.02 ± 9.71	92.03 ± 4.77	9.47 ± 0.09	20.51 ± 0.54	87.42 ± 8.46	2.63 ± 1.24
	20	41	3	25	21.67 ± 3.88	12.2 ± 0.7	1.18 ± 0.07	2.45 ± 0.03	75.85 ± 6.05	79.47 ± 5.21	8.70 ± 0.11	22.71 ± 1.11	79.22 ± 9.11	
	11	42	5	24	23.76 ± 3.06	12.9 ± 0.5	1.10 ± 0.05	2.34 ± 0.03	-	-	7.09 ± 0.09	17.87 ± 0.61	-	
Trask River	16	38	1	25	26.60 ± 3.42	13.0 ± 0.5	1.20 ± 0.07	2.57 ± 0.03	80.94 ± 5.96	96.10 ± 5.14	6.99 ± 0.10	17.59 ± 0.33	96.51 ± 10.52	3.93 ± 1.32
	20	46	6	25	24.41 ± 5.69	12.7 ± 0.8	1.19 ± 0.08	2.55 ± 0.03	78.00 ± 5.23	103.57 ± 6.36	8.11 ± 0.09	21.80 ± 1.18	112.00 ± 15.47	
	11	34	2	23	20.91 ± 4.03	12.4 ± 0.8	1.10 ± 0.05	2.29 ± 0.03	-	-	8.96 ± 0.10	18.78 ± 0.93	-	
Trinity River	16	39	0	25	23.65 ± 6.24	12.6 ± 1.1	1.15 ± 0.08	2.33 ± 0.02	94.24 ± 4.88	89.40 ± 4.62	7.79 ± 0.10	18.82 ± 0.46	84.56 ± 8.58	4.81 ± 2.56
	20	36	8	25	23.70 ± 4.42	12.6 ± 0.7	1.18 ± 0.09	2.40 ± 0.03	79.46 ± 4.63	76.22 ± 6.12	7.13 ± 0.19	23.59 ± 2.50	74.32 ± 11.71	

Fish swum is the number of successful swims conducted while mort. is the number of fish that did not survive the swimming trial or 24-hour recovery period after the trial. Maximum test temperature is the highest temperature at which fish could be successfully tested. Mass, fork length and condition factor (Fulton’s Cond. Factor) are reported as means and standard deviation of the observed data. AS and T_opt_ values are reported as mean and standard deviation of the model estimate calculated from repeated draws from the respective posterior distributions from the respective Bayesian models. For each metabolic rate (RMR, MMR, AS), we calculated the percentage of the 11°C acclimated rate observed at 16 or 20°C based upon the respective Bayesian models. Q_10_ coefficients of the RMR were calculated for each treatment (Q_10_). For each metabolic rate, we compared the mean percentage and standard deviation of metabolic capacity relative to a hatchery’s metabolic capacity when acclimated to 11°C. As metabolic rates were measured at acute test temperatures ranging from 11 to 26°C, we report the mean and standard deviation of the maintained percent across this range.

### Environmental associations with metabolic traits

Associations between environmental predictors and metabolic trait (RMR, MMR or AS) varied among thermal acclimation groups (11, 16 and 20°C). We highlight associations with migration distance (Mig.D) and the maximum temperature experienced during peak rearing (CRCMax) in Fig. 4. Other environmental associations are described further below for each metabolic trait.

**Figure 5:**
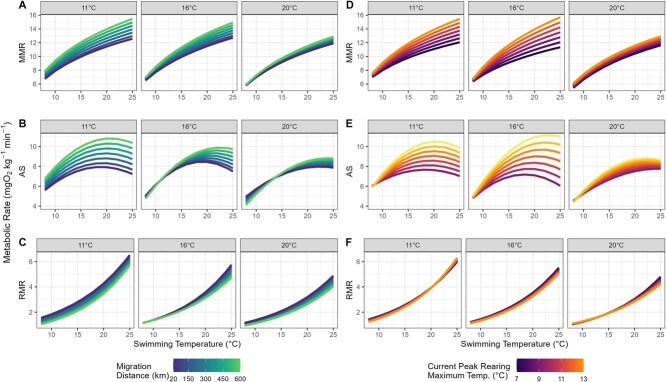
Statistical associations of environmental characteristics on metabolic traits of Fall-run Chinook Salmon across test temperatures. The association between test temperature and three metabolic traits MMR (A and D), AS (B and E) and RMR (C and F). Colors represent the modeled metabolic performance according to different hypothetical values of migration distances (Mig.D, A–C) or maximum temperature during peak juvenile rearing (CRCMax, D–F).

We identified strong significant negative associations between RMR and four environmental predictors (HAMax, CAMax, HARange and Mig.D). HAMax and Mig.D (Fig. 5C) were the only predictors to be strongly significant at multiple acclimation temperatures. When fish were acclimated to 11 or 20°C both traits were significantly negatively associated with RMR across test temperatures (Supplementary Table S12). RMR was similar across CRCMax and was not statistically significant; instead, RMR decreased with increasing acclimation temperatures (Fig. 4E).

Six environmental predictors had strong significant positive associations with MMR when fish were acclimated to 11 or 16°C, no predictors had strong significant associations when fish were acclimated to 20°C (Supplementary Table S13). These associations were strongest at warmer test temperatures. Significant environmental predictors included measurements of the current temperature regime, both annually and specific to periods of core juvenile rearing (CAMax, CARange, CRCMax, CRMax). Mig.D was positively associated with MMR when fish were acclimated to 11°C, and a weak, positive association was found among fish acclimated to 16°C (Fig. 5A). CRCMax was positively associated with MMR. The strength of this association increased with test temperature and was strongest among fish acclimated to 11°C or 16°C (Fig. 5D).

Among fish acclimated to 11 or 16°C, seven environmental predictors (Mig.D [Fig. 5B], CRCMax [Fig. 5E], CAMax, HAMax, CARange, HARange, and CRAve) had strongly significant positive associations with AS. Associations were strongest among fish acclimated to 11°C, and within acclimation groups the strength of the effect increased with test temperature. No environmental predictors were found to have strong significant associations with AS when fish were acclimated to 20°C (Supplementary Table S14).

## Discussion

Understanding the drivers of phenotypic variation among populations provides insight into how populations may respond to environmental change. Salmonids are broadly considered coldwater fish and increasing water temperatures associated with anthropogenic change continue to detrimentally affect these species (Moyle *et al.,* 2017). As such, populations occupying the warmest habitats may be expected to be at greater risk than populations from cooler habitats, but this assumption clouds the likelihood that responses will depend on the physiology of specific populations paired with local conditions. For instance, model estimated temperatures (Supplementary Table S8) indicate that the Priest Rapids population (WA) experiences greater annual maximum temperatures than the Coleman and Feather River populations (CA) yet exhibits slower growth rates when acclimated to 20°C. Furthermore, our results also indicate that longer-migrating populations (e.g. Coleman, Priest Rapids) may be metabolically limited as temperatures warm and therefore more at risk than short-migrating populations (e.g. Trask River, Elk River). Our research not only quantified population-specific physiological performance and variation in five traits but tested associations of this variation with 15 environmental predictors.

### Acute thermal tolerance is associated with local temperature characteristics

Across all six populations, CT_max_ values increased with acclimation temperature and were consistent with previous work on salmonids (Cech and Myrick, 1999; Myrick and Cech, 2000, 2001; Chen *et al.,* 2015). Unlike research on Brook Trout (*S. fontinalis* [Stitt *et al.,* 2014]) or Killifish (*Fundulus heteroclitus* [Fangue *et al.,* 2009]), we found weakly-significant and equivocal effects of latitude on CT_max_ a result that mirrors cross-taxa metanalysis on freshwater ectotherms (Sunday *et al.,* 2019). This result suggests that latitude may be a poor predictor of acute thermal tolerance, especially if local watershed characteristics (e.g. snowmelt-fed vs. rain-fed systems) disrupt the latitude/temperature gradient. In agreement with our hypothesis of local adaptation, we found strong significant associations between CT_max_ and aspects of environmental temperatures indicating populations from warmer habitats exhibit higher acute thermal maxima. Furthermore, our results are consistent with research on Fraser River (British Columbia, Canada) juvenile Sockeye Salmon (Chen *et al.,* 2013), identifying a weak negative association between migration distance and CT_max_. These results may be due to spatial autocorrelation on the landscape, whereby longer-migrations typically lead to higher elevations and therefore colder headwaters. Effects of local environmental traits and overall interpopulation differences were greatest when fish were warm-acclimated. It may be that wild fish acclimatized to cool environment (~11°C) are unlikely to experience stressful temperatures unexpectedly, but extended exposure to 16°C or 20°C cue fish to be physiologically prepared for additional thermal stress, manifesting population-specific, locally adapted thermal tolerance traits.

### Growth rate does not exhibit countergradient variation

Our results are inconsistent with a hypothesis of countergradient variation, wherein higher latitude populations are predicted to exhibit relatively faster growth at warm temperatures (Conover and Present, 1990; Sinnatamby *et al.,* 2015). Instead, the two southernmost populations (Coleman and Feather River) exhibited the fastest growth rate at 20°C. Furthermore, growth rate was positively associated with aspects of the local thermal environment, particularly traits capturing the maximum and average temperatures of habitats during the time of juvenile rearing, supporting a hypothesis that juvenile Chinook Salmon are locally adapted to their natal reaches and consistent with research demonstrating warm-adaptation among southern salmonid populations (Chen *et al.,* 2015; Verhille *et al.,* 2016; Poletto *et al.,* 2017). However, if fish were locally adapted, populations from colder habitats may be expected to grow faster at cooler temperatures relative to populations from warmer habitats. Our data does not support this. It may be that our coldest acclimation temperature (11°C) was not cold enough to elicit population-specific variations in coldwater physiology. Research on variation in coldwater tolerance might be improved by comparing the populations in this study to those from Alaska (USA) or eastern Russia. More northerly populations also have a shorter growing season. Therefore, countergradient variation, as observed in high-latitude (56–82°N) populations of Brook Trout (Sinnatamby *et al.,* 2015), may be detectable if studied populations extended to this northern extreme.

The Trinity River population exhibited the slowest growth of any population at any acclimation temperature, possibly due to differences in life-history (Beckman *et al.,* 1998). The Trinity River fall-run exhibiting three distinct outmigration strategies, whereas the rest of our studied populations predominantly exhibit one (Sullivan, 1989; Moyle *et al.,* 2017). The dominant life history strategy outmigrates during their first spring, similar to Fall-run populations elsewhere (Yoshiyama *et al.,* 1998; Moyle, 2002). A second life history strategy delays outmigration to the fall, oversummering in freshwater, while a third strategy entails juveniles spending an entire year in freshwater and outmigrating during the following spring. The slow growth of the Trinity River population we studied may reflect one of these delayed outmigration strategies (Beckman *et al.,* 1998). Extended freshwater residence has also been observed in the Elk River population, as well as the nearby population from the Sixes River (Oregon, USA) (Reimers, 1971, 1979). Mechanistic determinants of life-history strategies are unknown, and may be a product of hatchery production (McDonald *et al.,* 1998), hybridization with sympatric spring-run Chinook Salmon (Kinziger *et al.,* 2008), or an effect of captive rearing during the experiment. Diversity in outmigration timing, specifically late-outmigration, can buffer populations from extreme climatic events (Cordoleani *et al.,* 2021). Future work should explore the drivers of life-history diversity among Trinity River Chinook Salmon and the influence of hatchery practices on intra-population variation.

### Metabolic performance is suited to local environmental conditions

Metabolic performance was also consistent with local adaptation among populations. Higher environmental temperatures were positively associated with greater aerobic capacity (Supplementary Table S14), particularly when fish were acutely exposed to warmer test temperatures (20°C). These results are consistent with Eliason *et al.* (2011), indicating that metabolic traits may be locally adapted, as populations from warmer waters exhibited greater metabolic capacity when acutely tested under warm water conditions. However, these effects disappeared when populations were acclimated to 20°C, reflecting the shared decline in MMR and AS across populations. Reduced metabolic capacity under warm rearing conditions may reduce disease tolerance (Lapointe *et al.,* 2014; Bruneaux *et al.,* 2017), increase risk of predation (McInturf *et al.,* 2022) and indicates that despite maintaining aerobic performance at temperatures exceeding 23°C, juvenile Chinook Salmon remain cold-water fish. We were only able to include fish that survived the metabolic trials, and fish exposed to temperatures exceeding 23°C often succumbed to heat stress during the RMR (Supplementary Table S1) Therefore, our metabolic estimates of juvenile Chinook Salmon at warm temperatures may overestimate the true performance, as less thermally robust fish could not be represented.

We hypothesized that locally adapted metabolic traits may reflect the aerobic burden of the outmigration route, a response observed among adult salmonids but unknown among juveniles. Work by Eliason *et al.* (2011) found that populations of adult Sockeye Salmon from more challenging migratory environments exhibited greater metabolic and cardiac scopes and larger hearts. Micheletti *et al.* (2018) identified migration distance and migration slope as environmental predictors associated with genetic indicators of local adaptation among Steelhead Trout. We predicted that juveniles from populations undertaking longer, and more challenging migrations may require increased aerobic capacity. We found longer migration distances were significantly associated with lower RMR and greater MMR and AS, consistent with local adaptation. However, these associations were dependent upon acclimation temperature and disappeared with acclimation to 16 and 20°C. This result highlights the risks of future environmental warming; if adapted metabolic performance is eroded by warming temperatures then inland populations with long migrations may lack the aerobic capacity to complete their life-history strategies.

### Historical vs. current temperature predictions

Past work across taxa has indicated thermal physiology, especially heat tolerance, to be evolutionarily rigid ([Bibr ref6][Bibr ref6]; Hoffmann *et al.,* 2013; Sandblom *et al.,* 2016; Bennett *et al.,* 2021). Metabolic traits have evolved over long time scales to exploit the historical means of natural thermal regimes. In areas where current temperatures do not mirror historical regimes (e.g. highly modified waterways), a mismatch between current temperatures and metabolic traits may exist. Research on Sockeye Salmon in the dammed Fraser River system demonstrated that metabolic traits could be more strongly affiliated with historical temperatures as compared to current regimes (Eliason *et al.,* 2011). Our comparison of current (i.e. below-dam) and historical (i.e. above-dam) estimates of river temperature permit a coarse assessment of trait plasticity. Strongly significant associations between environmental temperature and CT_max_ were predominantly historical, consistent with a meta-analysis by Bennett *et al.* (2021) and the hypothesis of ‘concrete ceilings’ (Sandblom *et al.,* 2016), which posit that maximum thermal tolerances evolve slowly. However, associations with growth rate were balanced between current and historical estimates and associations with metabolic traits were mixed; RMR was more likely to be associated with historical temperatures and MMR and AS more commonly associated with current temperatures. Different responses among traits may indicate that adaptive rates in thermal performance are trait dependent, and that multiple physiological traits should be assessed when prescribing management or conservation criteria (Zillig *et al.,* 2021).

### Inter- and Intra-population variation changes with temperature

Inter- and intra-population variation in CT_max_ and growth rate were greatest at 20°C, a presumably more stressful condition for coldwater species. Stressed-induced phenotypic variation is widely observed (Rutherford and Lindquist, 1998; Queitsch *et al.,* 2002) and is hypothesized to be in part due to temperature-induced reduction of the efficacy of heat shock proteins that subsequently release cryptic genetic variation and ultimately phenotypic variation (Rutherford, 2000, 2003; Ghalambor *et al.,* 2007). Our results suggest that in a warmer future, populations of Chinook Salmon may express divergent phenotypes, which are hidden under historically natural temperature conditions (e.g. 11°C). Given discussions of genetic rescue (Robinson *et al.,* 2017) or population translocation (Lusardi and Moyle, 2017; Weise *et al.,* 2020), determination of population-specific thermal physiology acclimatized to future climate scenarios is necessary to identify populations most at risk or most robust (Gayeski *et al.,* 2018; Zillig *et al.,* 2021).

Counter to patterns among CT_max_ and growth rate, acclimation to warmer temperatures was found to erode differences between populations among measures of metabolic performance, particularly AS and MMR. For instance, the loss of an association with migratory distance (Fig. 5) is a result of a general reduction across populations in MMR when acclimated to 20°C (Fig. 4), which made populations more similar. This inverse relationship between MMR or AS and acclimation temperature may be expected of a coldwater fish and observed in early-migrating populations of Chinook Salmon (Zillig *et al.,* 2023). Future work investigating interpopulation differences must be cognizant of these acclimatory-related changes in thermal physiology, as populations may appear distinct or not depending on the rearing conditions.

### Hatchery supplementation

All the populations used in this study were sourced from hatcheries and care should be taken extrapolating the results to wild fish. Research on domestication effects on salmonids has revealed rapid declines in reproductive capacity among hatchery produced or supplemented populations (Araki *et al.,* 2008). Possible drivers of these deleterious hatchery effects include hatchery conditions (Araki *et al.,* 2008; Satake and Araki, 2012), adaptive or acclimatory pressures in hatcheries (Woodworth *et al.,* 2002; Chittenden *et al.,* 2010), spawning and release management strategies (Lusardi and Moyle, 2017; Sturrock *et al.,* 2019) or proportion of hatchery fish within the wild population (Araki *et al.,* 2008). In the present study, the selected populations differ in many aspects of hatchery production (e.g. number of spawners, release strategies), and therefore differences between populations and associations with environmental predictors may be confounded with ‘hatchery selection’. Despite the potential impacts of hatchery production on the physiology of juvenile Chinook Salmon, the contribution of these hatcheries (greater than 90% in some instances; Barnett-Johnson *et al.,* 2007) to the wild populations makes it relevant to study them for population-specific thermal physiology. Furthermore, protecting remaining Chinook Salmon genetic diversity is essential to population resilience, enabling hedging against stochastic environmental conditions via the portfolio effect (Carlson and Satterthwaite, 2011). For example, a companion study investigating Chinook Salmon thermal physiology among seasonal life-history strategies identified considerable similarity in the CT_max_ and growth rates among two introgressed hatchery populations (Zillig *et al.,* 2023). The possible loss of trait diversity, whether due to genetic homogenization or convergent hatchery selection, underscores the value in documenting hatchery phenotypes as a necessary step to identifying wild populations possessing novel variation in thermal physiology.

### Population responses

Salmonids are broadly considered cold-water fish, and increasing water temperatures continue to have detrimental effects upon salmonid species (Moyle *et al.,* 2017). It may be assumed that southern populations are more at risk than northern counterparts, but this assumption clouds the likelihood that the response of salmonids will depend on the physiology of specific populations paired with local conditions. For instance, the Priest Rapids population (WA) experiences greater annual maximum temperatures than the Coleman and Feather River populations (CA) yet exhibits slower growth rates when acclimated to 20°C. Furthermore, our results indicate that longer-migrating populations (e.g. Coleman, Priest Rapids) may be metabolically limited as temperatures warm and at greater risk than short-migrating populations (e.g. Trask River, Elk River). Therefore, of the studied populations, the northernmost Priest Rapids population, paradoxically, may be the most thermally imperiled. Our study highlights the importance of intraspecies variation and demonstrates that care should be taken when extrapolating physiological performance from geographically proximal populations or surrogate species.

## Funding

This work was supported by the US Environmental Protection Agency (N.A.F., W912P7-15-P-0015); the US Fish and Wildlife Service and University of California (N.A.F., F17AC00491); and the University of California, Agricultural Experiment Station (N.A.F., 2098-H).

## Author Contributions Statement

Conceptualization: K.W.Z., R.A.L. and N.A.F.; data curation: K.W.Z. and A.M.F.; formal analysis: K.W.Z. and A.M.F.; funding acquisition: K.W.Z. and N.A.F.; investigation: K.W.Z., A.M.F. and R.A.L.; methodology: K.W.Z., A.M.F., D.E.C. and N.A.F.; project administration: K.W.Z., D.E.C. and N.A.F.; resources: D.E.C. and N.A.F.; software: K.W.Z. and A.M.F.; supervision: K.W.Z., and N.A.F.; validation: K.W.Z. and A.M.F.; visualization: K.W.Z. and A.M.F.; writing—original draft: K.W.Z. and A.M.F.; writing—review and editing: K.W.Z., A.M.F., R.A.L., D.E.C. and N.A.F.;

## Data Availability

Data for this work can be found in Dryad at https://doi.org/10.25338/B8P63W.

## Supplementary Material

Web_Material_coad044
