# Supplementary material for: Intraspecific variation among Chinook Salmon populations indicates physiological adaptation to local environmental conditions
Source: Conserv Physiol. 2023 Jun 20;11(1):coad044. doi: 10.1093/conphys/coad044 (PMC10281501; doi:10.1093/conphys/coad044)
Supplement: Web_Material_coad044 [file web_material_coad044.zip › Zillig_Con_Phys_Supplemental_Information_Final_4.25.23.pdf]

Supporting Information for

Intraspecific variation among Chinook salmon populations indicates physiological adaptation to local environmental conditions

Authors

Kenneth W. Zillig[1], Alyssa M. FitzGerald[2,3], Robert A. Lusardi[1,4], Dennis E. Cocherell[1] and Nann A. Fangue[1*]

Affiliations

[1] Department of Wildlife, Fish and Conservation Biology, University of California, Davis CA 95616, USA.
[2] Institute of Marine Sciences, University of California Santa Cruz, Santa Cruz, CA, 95064, USA.
[3] Fisheries Ecology Division, Southwest Fisheries Science Center, National Marine Fisheries Service, National Oceanic and Atmospheric Administration, Santa Cruz, CA, 95060, USA.
[4] Center for Watershed Sciences, University of California, Davis CA 95616, USA.

*Corresponding author: nafangue@ucdavis.edu

This PDF file includes:

Figures S1 to S3
Tables S1 to S15

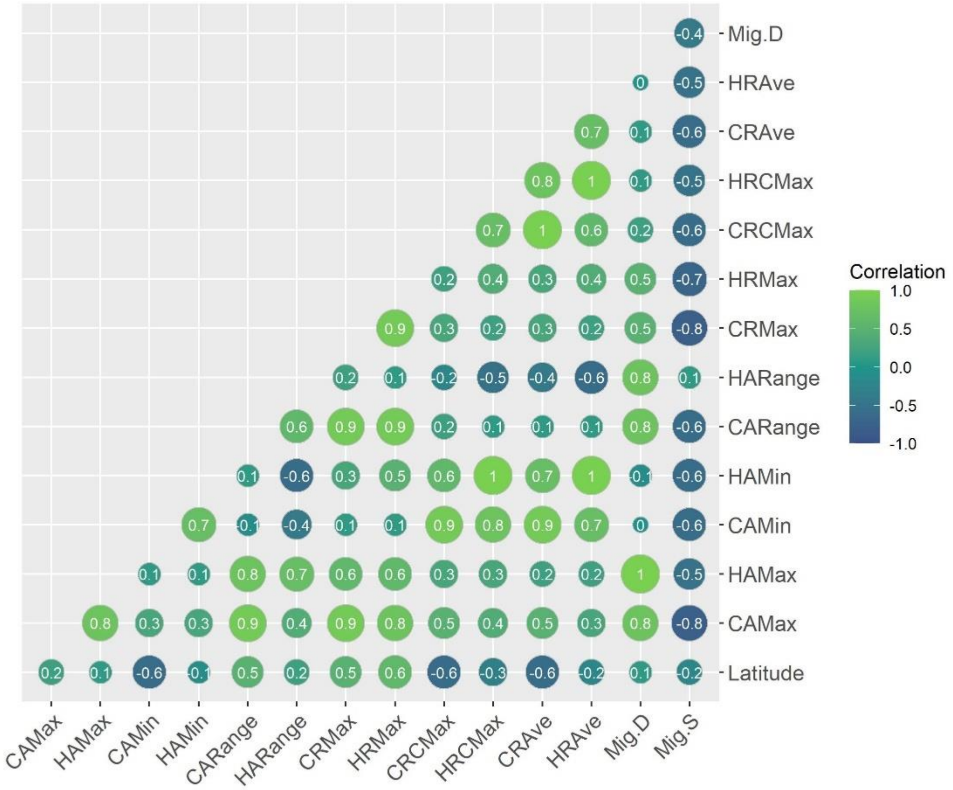

Fig. S1. Correlogram of associations of environmental predictor variables. The values represent the correlation among environmental predictors, while the size of the circle indicates the values distance from 0.

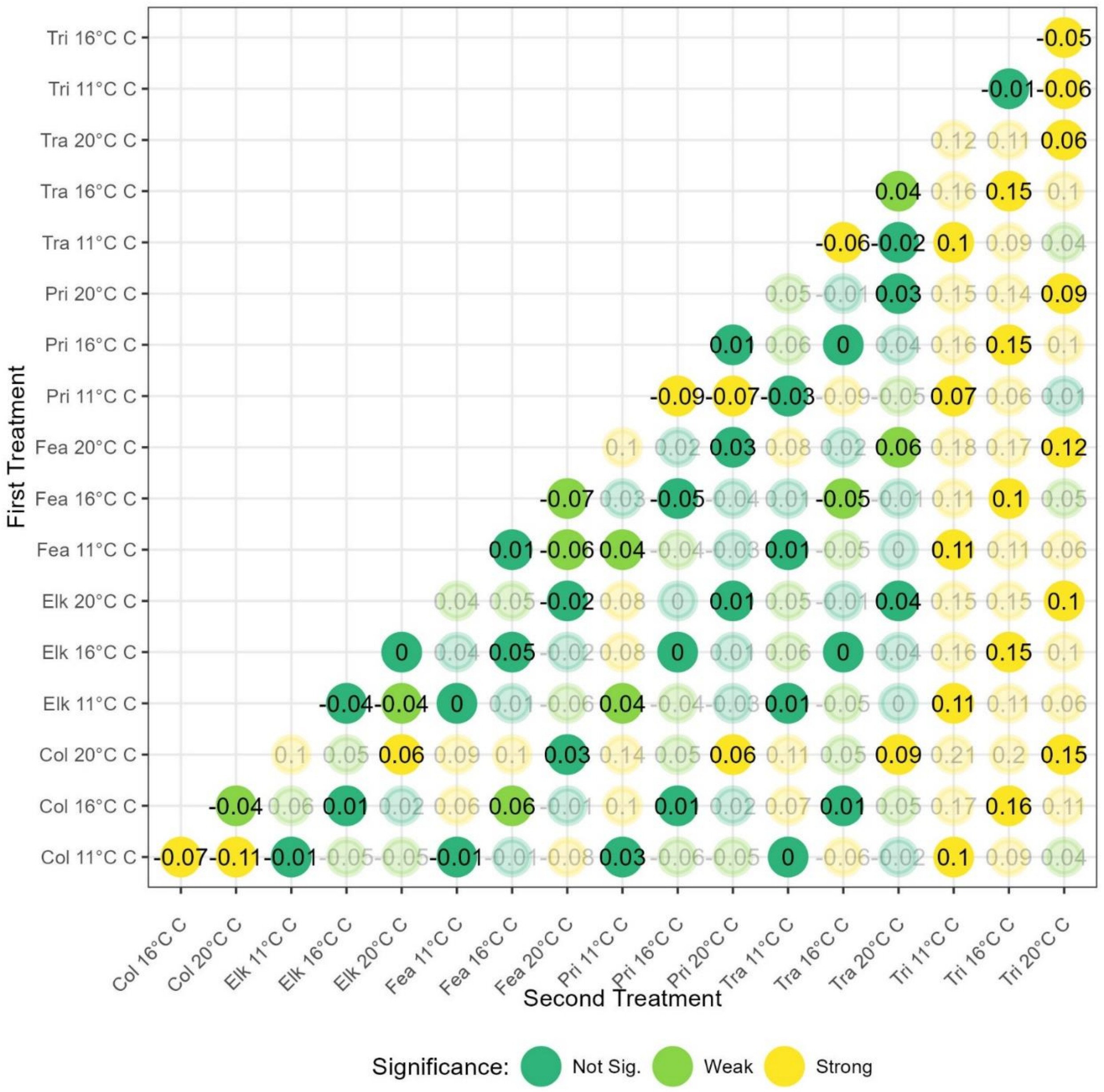

Fig. S2. Growth Rate treatment contrasts among six populations of Fall-run Chinook salmon reared at three temperatures. Col = Coleman, Fea = Feather River, Pri = Priest Rapids, Tri = Trinity River, Tra = Trask River, Elk = Elk River, 11°C, 16°C and 20°C indicate acclimation group. Using the lowest WAIC model the difference in growth rate between each treatment was estimated using the posterior distributions. The value upon each point is the mean difference in growth rate in (g/d) of the model-estimated growth rates of the First Treatment minus the Second Treatment. Point color identifies significance level; strong significance (yellow) was assigned if 94.5% of the contrast's posterior mass was above or below 0, weak significance (light green) was assigned if 85% of the posterior mass was above or below zero. Dark green indicates no assigned significance. Contrasts which share a single population or acclimation temperature are fully colored, while contrasts which do not share population or acclimation temperature are faded.

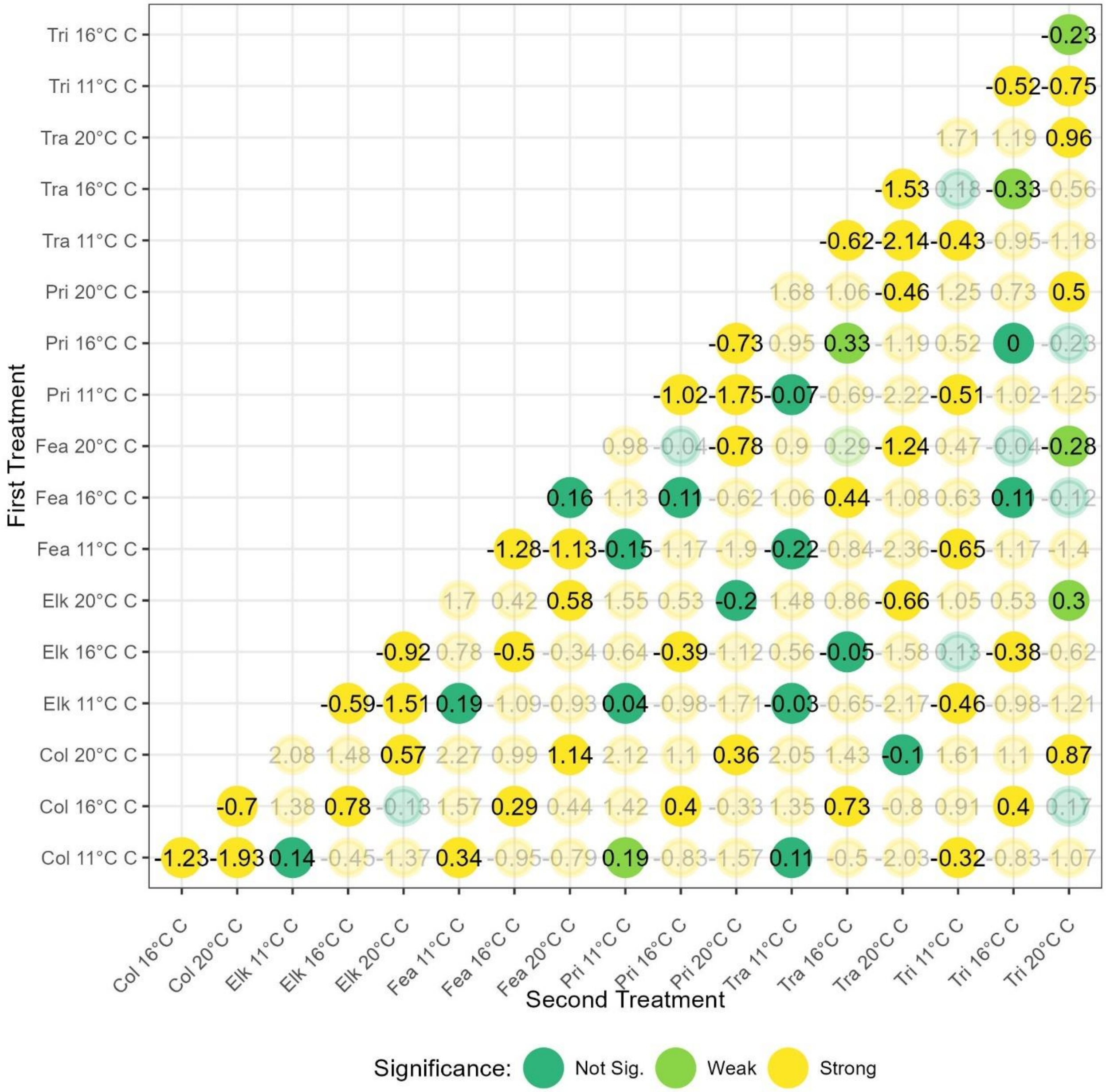

Fig. S3. Critical Thermal Maxima (CT_{max}) Treatment contrasts among six populations of Fall-run Chinook salmon reared at three temperatures. Col = Coleman, Fea = Feather River, Pri = Priest Rapids, Tri = Trinity River, Tra = Trask River, Elk = Elk River, 11°C, 16°C and 20°C indicate acclimation group. Using the lowest WAIC model the difference in CT_{max} between each treatment was estimated using the posterior distributions. The value upon each point is the mean difference in CT_{max} (°C) of the model-estimated growth rates of the First Treatment minus the Second Treatment. Point color identifies significance level; strong significance (yellow) was assigned if 94.5% of the contrast's posterior mass was above or below 0, weak significance (light green) was assigned if 85% of the posterior mass was above or below zero. Dark green indicates no assigned significance. Contrasts which are among a single population or acclimation temperature are fully colored, while contrasts which do not share population or acclimation temperature are faded.

Population	Acclimation Temp. (C°)	Trial Temp.	Fish (n)	Mass (g)	Fork Length (cm)	Condition Factor	Mort.
**Coleman**	11	8	3	21.71 ± 5.202	12.4 ± 0.74	1.13 ± 0.06	0
		10	3	22.80 ± 4.017	12.7 ± 0.57	1.11 ± 0.05	0
		12	4	21.90 ± 4.850	12.3 ± 0.57	1.17 ± 0.10	0
		14	3	21.36 ± 1.437	12.4 ± 0.44	1.12 ± 0.04	0
		16	3	21.25 ± 1.241	12.3 ± 0.10	1.14 ± 0.04	0
		18	4	22.78 ± 2.912	12.5 ± 0.48	1.16 ± 0.06	0
		20	4	20.88 ± 2.799	12.4 ± 0.50	1.09 ± 0.04	0
		22	4	24.78 ± 8.844	12.9 ± 1.29	1.12 ± 0.06	0
		24	8	21.78 ± 1.919	12.5 ± 0.92	1.12 ± 0.14	4
		25	1	NA	NA	NA	1
		26	2	NA	NA	NA	2
**Coleman**	16	8	5	21.48 ± 3.102	12.4 ± 0.58	1.12 ± 0.03	0
		10	6	23.74 ± 2.249	12.8 ± 0.48	1.14 ± 0.03	0
		12	5	22.70 ± 2.250	12.6 ± 0.51	1.12 ± 0.03	0
		14	4	24.09 ± 3.640	12.7 ± 0.61	1.18 ± 0.03	0
		16	4	24.80 ± 3.670	12.8 ± 0.57	1.18 ± 0.03	0
		18	4	23.71 ± 4.613	12.6 ± 0.71	1.17 ± 0.04	0
		20	3	22.18 ± 0.029	12.4 ± 0.10	1.16 ± 0.03	0
		22	4	25.25 ± 5.335	12.8 ± 0.60	1.20 ± 0.08	0
		24	4	25.85 ± 4.045	12.9 ± 0.60	1.21 ± 0.02	0
		25	4	23.75 ± 1.001	12.6 ± 0.06	1.20 ± 0.07	1
		26	2	NA	NA	NA	2
**Coleman**	20	8	4	28.49 ± 4.864	13.3 ± 0.44	1.20 ± 0.11	0
		10	4	26.37 ± 8.441	12.8 ± 1.31	1.23 ± 0.03	0
		12	5	26.05 ± 2.488	12.7 ± 0.44	1.26 ± 0.04	0
		14	5	25.75 ± 4.298	12.8 ± 0.59	1.23 ± 0.05	0
		16	6	21.48 ± 3.107	12.2 ± 0.78	1.18 ± 0.07	0
		18	4	21.71 ± 3.132	12.2 ± 0.54	1.19 ± 0.02	0
		20	5	25.82 ± 1.213	12.8 ± 0.12	1.23 ± 0.05	0
		22	4	24.69 ± 1.073	12.8 ± 0.30	1.17 ± 0.08	0
		24	4	23.43 ± 0.898	12.5 ± 0.28	1.19 ± 0.04	0
		25	4	24.13 ± 3.628	12.4 ± 0.60	1.26 ± 0.05	0
		26	3	NA	NA	NA	3
**Elk River**	11	8	4	27.44 ± 3.390	13.2 ± 0.65	1.20 ± 0.05	0
		10	4	29.73 ± 2.306	13.5 ± 0.41	1.22 ± 0.04	0
		12	6	28.82 ± 5.374	13.4 ± 0.67	1.20 ± 0.07	0
		14	4	23.56 ± 1.766	12.8 ± 0.17	1.13 ± 0.04	0
		16	4	28.01 ± 1.718	13.2 ± 0.19	1.22 ± 0.03	0
		18	4	27.54 ± 2.098	13.1 ± 0.34	1.24 ± 0.04	0
		20	5	23.98 ± 2.161	12.8 ± 0.45	1.14 ± 0.03	0
		22	4	26.22 ± 4.232	13.0 ± 0.67	1.19 ± 0.05	0
		24	7	24.78 ± 3.732	12.9 ± 0.70	1.16 ± 0.02	3
		25	2	NA	NA	NA	2

**Elk River**	16	8	4	24.66 ± 4.208	12.7 ± 0.72	1.21 ± 0.02	0
		10	5	22.41 ± 2.190	12.3 ± 0.58	1.19 ± 0.05	0
		12	3	24.68 ± 2.885	12.6 ± 0.72	1.23 ± 0.07	0
		14	4	24.00 ± 1.343	12.6 ± 0.37	1.22 ± 0.06	0
		16	4	23.97 ± 2.765	12.6 ± 0.39	1.20 ± 0.07	0
		18	5	22.55 ± 2.163	12.4 ± 0.30	1.19 ± 0.07	0
		20	4	24.53 ± 0.818	12.6 ± 0.26	1.23 ± 0.06	0
		22	5	23.34 ± 4.296	12.5 ± 0.80	1.20 ± 0.13	0
		24	5	24.78 ± 4.689	12.7 ± 0.66	1.19 ± 0.06	0
		25	2	NA	NA	NA	2
**Elk River**	20	8	5	26.93 ± 1.596	12.7 ± 0.38	1.33 ± 0.13	0
		10	4	27.48 ± 2.362	12.8 ± 0.34	1.30 ± 0.04	0
		12	5	27.07 ± 2.791	12.6 ± 0.51	1.35 ± 0.20	0
		14	5	24.00 ± 1.254	12.4 ± 0.50	1.26 ± 0.09	1
		16	4	22.82 ± 1.983	12.1 ± 0.18	1.29 ± 0.06	0
		18	4	23.09 ± 2.039	12.3 ± 0.33	1.25 ± 0.04	0
		20	6	25.86 ± 4.669	12.8 ± 0.50	1.22 ± 0.09	2
		22	4	25.90 ± 4.118	12.5 ± 0.70	1.32 ± 0.12	0
		24	5	25.11 ± 3.408	12.5 ± 0.52	1.28 ± 0.06	0
		25	7	24.38 ± 3.106	12.3 ± 0.51	1.30 ± 0.10	2
		26	2	NA	NA	NA	2
**Feather River**	11	8	4	23.53 ± 2.729	12.7 ± 0.32	1.14 ± 0.05	0
		10	6	25.51 ± 2.599	13.2 ± 0.43	1.11 ± 0.03	0
		12	4	26.22 ± 2.817	13.0 ± 0.41	1.19 ± 0.07	0
		14	4	24.31 ± 3.586	13.0 ± 0.60	1.11 ± 0.02	0
		16	4	24.56 ± 1.163	12.9 ± 0.24	1.15 ± 0.07	0
		18	4	25.45 ± 1.405	12.8 ± 0.29	1.21 ± 0.02	0
		20	5	26.11 ± 2.897	13.2 ± 0.61	1.14 ± 0.04	0
		22	4	25.81 ± 2.321	13.2 ± 0.22	1.12 ± 0.07	0
		23	4	26.48 ± 3.665	13.3 ± 0.66	1.13 ± 0.04	0
		24	2	NA	NA	NA	2
		25	2	NA	NA	NA	2
**Feather River**	16	8	4	25.38 ± 3.828	12.8 ± 0.59	1.21 ± 0.03	0
		10	4	26.55 ± 3.280	12.8 ± 0.38	1.26 ± 0.06	0
		12	4	23.10 ± 2.067	12.5 ± 0.17	1.20 ± 0.07	0
		14	4	22.38 ± 2.402	12.3 ± 0.35	1.22 ± 0.08	0
		16	4	24.29 ± 2.272	12.9 ± 0.36	1.13 ± 0.04	0
		18	4	24.53 ± 3.461	12.8 ± 0.34	1.16 ± 0.07	0
		20	4	23.00 ± 1.433	12.7 ± 0.26	1.12 ± 0.04	1
		22	4	23.64 ± 1.222	12.8 ± 0.28	1.12 ± 0.03	0
		24	6	23.67 ± 1.788	12.7 ± 0.39	1.15 ± 0.04	2
		25	2	NA	NA	NA	2
**Feather River**	20	8	6	27.43 ± 8.765	12.6 ± 1.03	1.34 ± 0.12	1
		10	5	28.41 ± 3.991	12.7 ± 0.23	1.38 ± 0.15	0

		12	4	23.70 ± 3.536	12.2 ± 0.13	1.32 ± 0.19	0
		14	5	23.58 ± 1.810	12.5 ± 0.33	1.21 ± 0.08	1
		16	4	26.11 ± 2.283	12.8 ± 0.29	1.26 ± 0.11	0
		18	4	25.20 ± 1.410	12.7 ± 0.37	1.23 ± 0.09	0
		20	4	24.68 ± 3.467	12.5 ± 0.34	1.27 ± 0.08	0
		22	4	28.31 ± 4.113	12.8 ± 0.42	1.36 ± 0.10	0
		24	7	27.57 ± 2.317	12.7 ± 0.45	1.36 ± 0.03	4
		25	5	22.71	12.2	1.25	4
		26	2	NA	NA	NA	2
**Priest Rapids**	11	8	4	21.26 ± 3.476	12.6 ± 0.58	1.07 ± 0.03	0
		10	4	19.63 ± 3.113	12.3 ± 0.66	1.05 ± 0.02	0
		12	4	16.10 ± 0.918	11.5 ± 0.17	1.07 ± 0.04	0
		14	6	22.71 ± 3.597	12.7 ± 0.67	1.10 ± 0.02	0
		16	6	22.04 ± 4.318	12.6 ± 0.69	1.10 ± 0.06	0
		18	4	22.11 ± 2.692	12.8 ± 0.33	1.06 ± 0.05	0
		20	4	21.40 ± 4.940	12.7 ± 0.83	1.04 ± 0.05	0
		22	5	19.24 ± 5.461	12.1 ± 0.93	1.07 ± 0.04	0
		24	4	19.51 ± 3.416	12.2 ± 0.54	1.07 ± 0.05	0
		25	2	NA	NA	NA	2
**Priest Rapids**	16	8	6	26.68 ± 3.053	13.2 ± 0.20	1.16 ± 0.09	0
		10	4	21.36 ± 2.448	12.6 ± 0.32	1.07 ± 0.08	0
		12	4	20.37 ± 2.238	12.1 ± 0.25	1.14 ± 0.06	0
		14	4	25.53 ± 3.427	12.9 ± 0.30	1.19 ± 0.07	0
		16	4	19.41 ± 2.497	12.1 ± 0.65	1.11 ± 0.04	0
		18	4	25.27 ± 2.362	13.0 ± 0.39	1.15 ± 0.01	0
		20	4	22.77 ± 3.176	12.6 ± 0.48	1.15 ± 0.05	0
		22	5	20.70 ± 1.354	12.4 ± 0.27	1.07 ± 0.04	0
		24	5	21.46 ± 2.559	12.4 ± 0.36	1.13 ± 0.05	0
		25	3	NA	NA	NA	3
		26	2	NA	NA	NA	2
**Priest Rapids**	20	8	4	23.13 ± 2.352	12.5 ± 0.51	1.18 ± 0.05	0
		10	4	28.21 ± 2.853	13.2 ± 0.33	1.24 ± 0.05	0
		12	4	23.42 ± 2.418	12.6 ± 0.50	1.17 ± 0.06	0
		14	4	19.44 ± 0.777	11.8 ± 0.30	1.19 ± 0.05	0
		16	4	19.040 ± 1.863	11.7 ± 0.26	1.18 ± 0.11	0
		18	3	23.02 ± 4.496	12.6 ± 0.71	1.13 ± 0.03	0
		20	5	18.50 ± 2.001	11.8 ± 0.36	1.13 ± 0.05	0
		22	5	24.46 ± 3.389	12.7 ± 0.55	1.18 ± 0.02	0
		24	4	19.91 ± 3.001	11.9 ± 0.33	1.18 ± 0.09	0
		25	5	17.99 ± 0.143	11.5 ± 0.31	1.20 ± 0.10	1
		26	2	NA	NA	NA	2
**Trask River**	11	8	4	22.37 ± 0.794	12.6 ± 0.13	1.13 ± 0.06	0
		10	6	23.39 ± 4.748	13.0 ± 0.70	1.07 ± 0.06	0
		12	4	24.85 ± 1.564	13.1 ± 0.29	1.10 ± 0.01	0

		14	4	22.71 ± 3.287	12.7 ± 0.61	1.12 ± 0.02	0
		16	5	24.35 ± 4.010	13.0 ± 0.62	1.11 ± 0.05	0
		18	4	23.95 ± 2.157	12.9 ± 0.42	1.11 ± 0.06	0
		20	6	24.59 ± 4.029	13.1 ± 0.62	1.10 ± 0.06	0
		22	6	23.88 ± 2.458	12.9 ± 0.34	1.10 ± 0.05	1
		24	6	23.36 ± 2.673	12.7 ± 0.39	1.13 ± 0.03	2
		25	2	NA	NA	NA	2
**Trask River**	16	8	4	30.45 ± 4.612	13.3 ± 0.53	1.28 ± 0.06	0
		10	3	26.09 ± 5.455	13.1 ± 0.60	1.15 ± 0.08	0
		12	3	24.74 ± 2.720	12.8 ± 0.46	1.19 ± 0.04	0
		14	4	28.15 ± 1.704	13.2 ± 0.32	1.23 ± 0.10	0
		16	4	26.13 ± 1.859	13.1 ± 0.46	1.16 ± 0.05	0
		18	4	27.64 ± 5.117	13.3 ± 0.70	1.16 ± 0.03	0
		20	4	23.83 ± 1.758	12.6 ± 0.20	1.19 ± 0.09	0
		22	4	25.92 ± 1.572	12.8 ± 0.41	1.24 ± 0.06	0
		24	4	24.33 ± 1.840	12.6 ± 0.38	1.21 ± 0.04	0
		25	5	28.10 ± 2.674	13.2 ± 0.56	1.21 ± 0.07	1
**Trask River**	20	8	4	23.29 ± 3.202	12.5 ± 0.59	1.20 ± 0.07	0
		10	4	26.06 ± 3.963	12.8 ± 0.72	1.23 ± 0.06	0
		12	4	22.27 ± 2.207	12.3 ± 0.33	1.21 ± 0.08	0
		14	6	31.82 ± 9.508	13.8 ± 1.34	1.18 ± 0.05	0
		16	4	26.38 ± 3.032	13.0 ± 0.47	1.20 ± 0.03	0
		18	4	21.83 ± 2.329	12.3 ± 0.43	1.18 ± 0.06	0
		20	5	24.07 ± 5.354	12.7 ± 0.82	1.17 ± 0.07	0
		22	6	20.58 ± 4.069	12.3 ± 0.71	1.09 ± 0.08	0
		24	7	22.18 ± 2.863	12.3 ± 0.40	1.19 ± 0.07	2
		25	6	24.49 ± 6.339	12.4 ± 0.57	1.27 ± 0.13	2
		26	2	NA	NA	NA	2
**Trinity River**	11	8	4	21.91 ± 4.448	12.7 ± 1.02	1.07 ± 0.04	0
		10	4	23.17 ± 5.121	12.8 ± 0.93	1.11 ± 0.07	0
		12	4	19.34 ± 4.270	12.0 ± 0.88	1.12 ± 0.03	0
		14	4	19.36 ± 3.315	12.3 ± 0.68	1.05 ± 0.04	0
		16	4	22.87 ± 6.106	12.7 ± 0.91	1.09 ± 0.08	0
		18	4	22.02 ± 3.189	12.6 ± 0.65	1.11 ± 0.04	0
		20	3	16.90 ± 1.313	11.5 ± 0.31	1.10 ± 0.02	0
		22	3	20.74 ± 3.841	12.2 ± 0.81	1.13 ± 0.03	0
		23	4	20.86 ± 2.694	12.4 ± 0.71	1.11 ± 0.05	0
		24	2	NA	NA	NA	2
**Trinity River**	16	8	4	29.83 ± 2.566	13.7 ± 0.50	1.17 ± 0.03	0
		10	3	28.06 ± 3.794	13.3 ± 0.50	1.20 ± 0.11	0
		12	4	25.92 ± 7.590	12.8 ± 1.29	1.21 ± 0.07	0
		14	4	21.90 ± 10.363	12.3 ± 1.70	1.10 ± 0.10	0
		16	4	18.38 ± 4.415	11.7 ± 1.13	1.13 ± 0.07	0
		18	4	22.28 ± 6.280	12.3 ± 1.28	1.18 ± 0.08	0

		20	4	23.85 ± 4.684	12.6 ± 0.75	1.17 ± 0.03	0
		22	4	19.69 ± 0.838	12.4 ± 0.30	1.05 ± 0.05	0
		24	4	22.51 ± 9.037	12.3 ± 1.62	1.17 ± 0.06	0
		25	4	25.18 ± 1.475	12.9 ± 0.19	1.18 ± 0.07	0
**Trinity River**	20	8	4	21.85 ± 3.699	12.2 ± 0.68	1.20 ± 0.04	0
		10	4	24.67 ± 4.182	12.6 ± 0.53	1.22 ± 0.05	0
		12	3	28.46 ± 1.401	13.4 ± 0.26	1.18 ± 0.05	0
		14	4	28.08 ± 3.250	13.1 ± 0.50	1.25 ± 0.09	0
		16	4	27.15 ± 1.280	13.1 ± 0.17	1.22 ± 0.10	0
		18	5	23.11 ± 5.636	12.8 ± 0.64	1.09 ± 0.13	1
		20	4	18.68 ± 1.511	11.7 ± 0.29	1.18 ± 0.09	0
		22	4	21.62 ± 3.896	12.4 ± 0.91	1.14 ± 0.07	0
		24	4	20.96 ± 3.797	12.2 ± 0.85	1.16 ± 0.08	0
		25	6	23.50	12.7	1.15	5
		26	2	NA	NA	NA	2

Table S1: Summary table of all populations and test temperatures. ‘Fish (n)’ is the total number of fish attempted while ‘Mort.’ Is the number of mortalities at each temperature. Mass, fork length and condition factor are reported as the mean and standard deviation of the observed fish that underwent the trial. Accurate weights and lengths could not be obtained for mortalities.

	Model	WAIC	ΔWAIC	w_i
Fish Growth	**Mass ~ 1 + Time*Pop.*A°C+Mass_t^0**	**5385.5**	**0**	**0.65613**
	**Mass ~ 1 + Time*Pop.*A°C+Mass_t^0+(1|Tank_ID)**	**5386.9**	**1.4**	**0.32740**
	Mass ~ 1 + Time*Pop.*A°C+Mass_t^0+(1+Pop.|Tank_ID)	5392.9	7.4	0.01618
	Mass ~ 1 + Time*Pop.*A°C+(1|Tank_ID)	5401.2	15.7	0.00004
	Mass ~ 1 + Time*Pop.*A°C+(1+Pop.|Tank_ID)	5401.2	15.7	0.00026
	Mass ~ 1 + Time*Pop.*A°C	5420.1	34.6	0.00000
	Mass ~ 1 + Time*Pop.	5486.8	101.3	0.00000
	Mass ~ 1 + Time*A°C	5671.8	286.3	0.00000
	Mass ~ 1 + Time	5682.6	297.1	0.00000
	Mass ~ 1	6102.3	716.8	0.00000

Table S2: Stepwise model selection for fish mass. Models with the lowest WAIC score were selected for further analysis. Growth rate was modeled as a function of Time, population (Pop.), acclimation temperature (A°C), initial mass (Mass_t^0) and a random effect for rearing tank (Tank_ID). There were two models (bolded) which were considered not statistically different.

	Model	WAIC	ΔWAIC	w_i
Critical Thermal Maximum	**CT_{max} ~ 1 + A°C*Pop.+A°C*Mass + DPH + (1|CTM_Chamber)**	**779.5**	**0**	**0.97286**
	CT_{max} ~ 1 + A°C*Pop. + Mass	788.2	8.7	0.01234
	CT_{max} ~ 1 + A°C*Pop. + Mass + DPH + (1|CTM_Chamber)	789.2	9.7	0.00733
	CT_{max} ~ 1 + A°C*Pop. + Mass + (1| CTM_Chamber)	789.7	10.2	0.00587
	CT_{max} ~ 1 + A°C*Pop.*Mass + DPH + (1|CTM_Chamber)	792.7	13.2	0.00133
	CT_{max} ~ 1 + A°C*Pop. + FL	797.4	17.9	0.00012
	CT_{max} ~ 1 + A°C*Pop. + FL + DPH + (1| CTM_Chamber)	798.3	18.8	0.00008
	CT_{max} ~ 1 + A°C*Pop. + FL + (1| CTM_Chamber)	798.8	19.3	0.00006
	CT_{max} ~ 1 + A°C*Pop. + C_Fac	810.2	30.7	0.00000
	CT_{max} ~ 1 + A°C*Pop. + C_Fac + (1|CTM_Chamber)	811.4	31.9	0.00000
	CT_{max} ~ 1 + A°C*Pop.	815.6	36.1	0.00000
	CT_{max} ~ 1 + A°C*Pop.*C_Fac	821.3	41.8	0.00000
	CT_{max} ~ 1 + A°C	896.7	117.2	0.00000
	CT_{max} ~ 1 + C_Fac	1065.3	285.8	0.00000
	CT_{max} ~ 1 + Pop.	1072.3	292.8	0.00000
	CT_{max} ~ 1 + Mass	1078.2	298.7	0.00000
	CT_{max} ~ 1	1079.3	299.8	0.00000

Table S3: Stepwise model selection for Critical Thermal Maxima (CT_{max}). Models with the lowest WAIC score were selected for further analysis. CT_{max} was modeled as a function of population (Pop.), acclimation temperature (A°C), Fulton's condition factor (C_Fac), fish age (DPH), Mass and random effects of CT_{max} test chamber (CTM_Chamber).

	Model	*WAIC*	*ΔWAIC*	*wi*
Routine Metabolic Rate	**LOG_RMR ~ 1 + T°C*Pop.*A°C+C_Fac+Tunnel_ID+DPH**	**294.1**	**0**	**0.50379**
	**LOG_RMR ~ 1 + T°C*Pop.*A°C+Tunnel_ID+DPH**	**294.3**	**0.2**	**0.45016**
	LOG_RMR ~ 1 + T°C*Pop.*A°C+C_Fac*A°C+Tunnel_ID+DPH	298.9	4.8	0.04604
	LOG_RMR ~ 1 + T°C*Pop.*A°C+Tunnel_ID	315.8	21.7	0.00000
	LOG_RMR ~ 1 + T°C*Pop.*A°C+DPH	319.9	25.8	0.00000
	LOG_RMR ~ 1 + T°C*Pop.*A°C	339.9	45.8	0.00000
	LOG_RMR ~ 1 + T°C*Pop.*A°C+Mass	340.3	46.2	0.00000
	LOG_RMR ~ 1 + T°C*Pop.*A°C+C_Fac	340.8	46.7	0.00000
	LOG_RMR ~ 1 + T°C*Pop.*A°C+Mass*A°C	342.5	48.4	0.00000
	LOG_RMR ~ 1 + T°C*Pop.*A°C+C_Fac*A°C	344.4	50.3	0.00000
	LOG_RMR ~ 1 + T°C*A°C	379.8	85.7	0.00000
	LOG_RMR ~ 1 + T°C*Pop.	624.7	330.6	0.00000
	LOG_RMR ~ 1 + T°C	642	347.9	0.00000
	LOG_RMR ~ 1	2017.6	1723.5	0.00000
Maximum Metabolic Rate	**MMR ~ 1 + Log(T°C)*Pop.*A°C + Tunnel_ID + C_Fac**	**1163.7**	**0**	**0.77724**
	MMR ~ 1 + Log(T°C)*Pop.*A°C + DPH + C_Fac + Tunnel_ID	1166.2	2.5	0.22266
	MMR ~ 1 + Log(T°C)*Pop.*A°C + Tunnel_ID + Mass	1182.0	18.3	0.00008
	MMR ~ 1 + Log(T°C)*Pop.*A°C + C_Fac	1184.4	20.7	0.00002
	MMR ~ 1 +Log(T°C)*Pop.*A°C + Tunnel_ID	1191.2	27.5	0.00000
	MMR ~ 1 + Log(T°C)*Pop.*A°C + Tunnel_ID + DPH	1193.1	29.4	0.00000
	MMR ~ 1 + Log(T°C)*Pop.*A°C	1213.2	49.5	0.00000
	MMR ~ 1 + T°C*Pop.*A°C + C_Fac	1218.6	54.9	0.00000
	MMR ~ 1 + T°C*Pop.*A°C + T°C^2*Pop.*A°C	1238.1	74.4	0.00000
	MMR ~ 1 + T°C*Pop.*A°C	1250.2	86.5	0.00000
	MMR ~ 1 + T°C*A°C + T°C*A°C	1397.8	234.1	0.00000
	MMR ~ 1 + T°C*Pop.+T°C^2*Pop.	1459.5	295.8	0.00000
	MMR ~ 1 + T°C + T°C^2	1529.0	365.3	0.00000
	MMR ~ 1 + T°C	1563.1	399.4	0.00000
	MMR ~ 1	2017.6	853.9	0.00000
Aerobic Scope	**AS ~ 1 + T°C*Pop.*A°C + T°C^2*Pop.*A°C + C_Fac*A°C + Tunnel_ID**	**1564.3**	**0**	**0.81051**
	AS ~ 1 + T°C*Pop.*A°C + T°C^2*Pop.*A°C + C_Fac*A°C + Tunnel_ID+DPH	1567.4	3.1	0.17118
	AS ~ 1 + T°C*Pop.*A°C + T°C^2*Pop.*A°C + C_Fac*A°C + Tunnel_ID + (1 + Pop.|Tank_ID)	1571.4	7.1	0.01626
	AS ~ 1 + T°C*Pop.*A°C + T°C^2*Pop.*A°C + C_Fac*A°C	1577.4	13.1	0.00121
	AS ~ 1 + T°C*Pop.*A°C + T°C^2*Pop.*A°C + C_Fac	1578.2	13.9	0.00084
	AS ~ 1 + T°C*Pop.*A°C + T°C^2*Pop.*A°C + Mass	1591.4	27.1	0.00000
	AS ~ 1 + T°C*Pop.*A°C + T°C^2*Pop.*A°C + Mass*A°C	1592.3	28	0.00000
	AS ~ 1 + T°C*Pop.*A°C + T°C^2*Pop.*A°C + DPH	1603.8	39.5	0.00000
	AS ~ 1 + T°C*Pop.*A°C	1659.7	95.4	0.00000
	AS ~ 1 + T°C*Pop.	1813.8	249.5	0.00000
	AS ~ 1 + T°C + T°C^2	1838.7	274.4	0.00000
	AS ~ 1 + T°C*A°C	1839.9	275.6	0.00000
	AS ~ 1 + T°C	1906.2	341.9	0.00000
	AS ~ 1	2017.9	453.6	0.00000

Table S4: Stepwise model selection for metabolic trait models. The natural log of Routine Metabolic Rate (RMR) was modeled as a function of population (Pop.), acclimation temperature (A°C), test temperature (T°C), Mass, Fulton's Condition Factor (C_Fac), fish age (DPH) and swim tunnel (Tunnel_ID). The lowest WAIC model (bolded) are the model used for determining results. In the case of multiple bolded models, the two

models are not statistically different from one another, but the lower WAIC model was used for further analysis. Maximum metabolic rate (MMR) was modeled as a function of both the natural log of test temperature (Log(T°C)) and a quadratic function of test temperature. Aerobic scope was modeled as a quadratic function of test temperature.

	Model	*WAIC*	*ΔWAIC*	w_i
Fish Growth	**Mass ~ 1 + Time*Lat*A°C + Mass_t^0 + (1|Hatchery) + (1|Tank_ID)**	**5444.6**	**0**	**0.77609**
	Mass ~ 1 + Time*Lat* A°C + Mass_t^0 + (1|Hatchery)	5447.6	3	0.16827
	Mass ~ 1 + Time* A°C + Mass_t^0 + (1|Hatchery) + (1|Tank_ID)	5449.9	5.3	0.05564
	Mass ~ 1 + Time*Lat* A°C + Mass_t^0	5506	61.4	0.00000
	Mass ~ 1 + Time*Lat* A°C	5645.3	200.7	0.00000
	Mass ~ 1 + Time*Lat	5669.5	224.9	0.00000
	Mass ~ 1 + Time* A°C	5671.7	227.1	0.00000
	Mass ~ 1 + Time	5682.5	237.9	0.00000
	Mass ~ 1 + A°C	6093.4	648.8	0.00000
	Mass ~ 1 + Lat	6101.1	656.5	0.00000
	Mass ~ 1	6102.4	657.8	0.00000

Table S5: Stepwise model selection for associations between growth rate and latitude. Models with the lowest WAIC score were selected for further analysis. Growth rate was modeled as a function of Time, latitude (Lat), acclimation temperature (A°C), initial mass (Mass_t^0) and random effects of Hatchery or rearing tank (Tank_ID).

	Model	*WAIC*	*ΔWAIC*	w_i
Critical Thermal Maximum	**CTmax~ 1 + A°C*Lat + C_Fac*A°C + DPH + (1|CTM_Chamber) + (1|Hatchery)**	**817.8**	**0**	**0.99998**
	CTmax~ 1 + A°C*Lat + C_Fac*A°C + DPH + (1|CTM_Chamber)	840.4	22.6	0.00001
	CTmax~ 1 + A°C*Lat + Mass*A°C	841.4	23.6	0.00000
	CTmax~ 1 + A°C*Lat + C_Fac*A°C + (1|CTM_Chamber)	842.8	25	0.00000
	CTmax~ 1 + A°C*Lat + Mass	853.6	35.8	0.00000
	CTmax~ 1 + A°C*Lat + C_Fac	868.6	50.8	0.00000
	CTmax~ 1 + A°C*Lat + C_Fac*A°C	869.4	51.6	0.00000
	CTmax~ 1 + A°C*Lat	879.7	61.9	0.00000
	CTmax~ 1 + A°C	896.7	78.9	0.00000
	CTmax~ 1	1079.3	261.5	0.00000
	CTmax~ 1 + Lat	1081.2	263.4	0.00000

Table S6: Stepwise model selection for associations between Critical Thermal Maximum (CTmax) and latitude. Models with the lowest WAIC score were selected for further analysis. CTmax was modeled as a function of latitude (Lat), acclimation temperature (A°C), Fulton's condition factor (C_Fac), fish age (DPH), Mass and random effects of Hatchery or CTM test chamber (CTM_Chamber).

	Model	WAIC	ΔWAIC	w_i
Routine Metabolic Rate	**Log(RMR) ~ 1 + T°C*A°C*Lat + C_Fac + Tunnel_ID + DPH + (1|Population)**	**314.4**	**0**	**0.98995**
	Log(RMR) ~ 1 + T°C*A°C*Lat + Tunnel_ID + (1|Population)	323.6	9.2	0.01005
	Log(RMR) ~ 1 + T°C*A°C*Lat+Tunnel_ID	363.7	49.3	0.00000
	Log(RMR) ~ 1 + T°C*A°C*Lat+ C_Fac + Tunnel_ID+DPH	365.5	51.1	0.00000
	Log(RMR) ~ 1 + T°C*A°C	379.8	65.4	0.00000
	Log(RMR) ~ 1 + T°C*A°C*Lat	385.1	70.7	0.00000
	Log(RMR) ~ 1 + T°C*A°C*Lat+ C_Fac	386.0	71.6	0.00000
	Log(RMR) ~ 1 + T°C*A°C*Lat+ DPH	386.8	72.4	0.00000
	Log(RMR) ~ 1 + T°C	642.0	327.6	0.00000
	Log(RMR) ~ 1	2017.6	1703.2	0.00000
Maximum Metabolic Rate	**MMR ~ 1 + Log(T°C)*Lat*A°C+DPH +Tunnel_ID+C_Fac+(1|Population)**	**1246.2**	**0**	**1.00000**
	MMR ~ 1 + Log(T°C)*Lat*A°C+DPH+Tunnel_ID+C_Fac	1297.3	51.1	0.00000
	MMR ~ 1 + Log(T°C)*Lat*A°C+C_Fac	1354.0	107.8	0.00000
	MMR ~ 1 + Log(T°C)*Lat*A°C+DPH	1357.0	110.8	0.00000
	MMR ~ 1 + Log(T°C)*Lat*A°C+Tunnel_ID	1375.3	129.1	0.00000
	MMR ~ 1 + Log(T°C)*Lat*A°C	1392.8	146.6	0.00000
	MMR ~ 1 + Log(T°C)	1536.6	290.4	0.00000
	MMR ~ 1 + Log(T°C)*Lat	1539.1	292.9	0.00000
	MMR ~ 1	2017.6	771.4	0.00000
Aerobic Scope	**AS ~ 1 + Lat*A°C*T°C + Lat*A°C*T°C^2 + C_Fac * A°C + Tunnel_ID + (1|Population)**	**1624.0**	**0**	**1.00000**
	AS ~ 1 + Lat*A°C*T°C + Lat*A°C*T°C^2 + C_Fac* A°C + Tunnel_ID	1715.7	91.7	0.00000
	AS ~ 1 + Lat*A°C*T°C + Lat*A°C*T°C^2	1766.9	142.9	0.00000
	AS ~ 1 + Lat*A°C*T°C	1830.9	206.9	0.00000
	AS ~ 1 + T°C + T°C^2	1838.7	214.7	0.00000
	AS ~ 1 + A°C*T°C	1839.9	215.9	0.00000
	AS ~ 1 + T°C	1906.2	282.2	0.00000
	AS ~ 1 + Lat*T°C	1906.5	282.5	0.00000
	AS ~ 1	2017.9	393.9	0.00000

Table S7: Stepwise model selection for associations between metabolic traits and latitude. The natural log of Routine Metabolic Rate (RMR) was modeled as a function of latitude (Lat), acclimation temperature (A°C), test temperature (T°C), Mass, Fulton’s Condition Factor (C_Fac), fish age (DPH) and swim tunnel (Tunnel_ID). There were additional random effects for Population The two models with the lowest WAIC (bolded) were non statistically different. Maximum metabolic rate (MMR) was modeled as a function of both the natural log of test temperature (Log(T°C)) and a quadratic function of test temperature. Aerobic scope was modeled as a quadratic function of test temperature.

	Annual Max		Annual Min		Annual Range		Rearing Average		Core Rearing Max		Rearing Max		Migration Distance & Slope	
	C	H	C	H	C	H	C	H	C	H	C	H	km	m/km
**Population**	CAMAX	HAMAX	CAMIN	HAMIN	CARANGE	HARANGE	CRAVE	HRAVE	CRCMAX	HRCMAX	CRMAX	HRMAX	MIG.D	MIG.S
Coleman	21.0 ± 0.49	19.6 ± 0.52	8.2 ± 0.12	8.1 ± 0.10	12.8 ± 0.55	11.5 ± 0.48	10.7 ± 0.14	10.9 ± 0.26	12.3 ± 0.18	12.5 ± 0.23	17.8 ± 0.57	17.2 ± 0.21	441.4	0.72
Elk River	18.0 ± 0.13	16.7 ± 0.3	6.5 ± 0.05	6.2 ± 0.12	11.5 ± 0.13	10.5 ± 0.21	9.4 ± 0.12	8.6 ± 0.19	9.8 ± 0.13	9.0 ± 0.21	18.0 ± 0.13	16.7 ± 0.30	21.9	4.14
Feather River	20.4 ± 2.10	17.3 ± 0.96	8.2 ± 0.37	5.7 ± 0.48	12.2 ± 2.46	11.6 ± 0.77	11.1 ± 0.36	7.4 ± 0.78	13.0 ± 0.68	8.7 ± 0.91	19.2 ± 1.49	15.3 ± 1.05	232.7	0.46
Priest Rapids	23.3 ± 0.56	20.4 ± 0.48	6.0 ± 0.17	5.0 ± 0.23	17.3 ± 0.41	15.5 ± 0.26	8.3 ± 0.3	6.2 ± 0.45	9.3 ± 0.31	7.0 ± 0.47	23.0 ± 0.43	20.4 ± 0.48	630.7	0.51
Trask River	17.3 ± 0.36	16.0 ± 0.12	6.6 ± 0.06	6.4 ± 0.07	10.7 ± 0.34	9.6 ± 0.13	8.2 ± 0.12	7.6 ± 0.08	8.6 ± 0.15	8.0 ± 0.07	17.3 ± 0.36	16.0 ± 0.12	28.2	1.56
Trinity River	15.8 ± 1.89	17.3 ± 1.68	5.8 ± 0.43	3.3 ± 0.36	10.0 ± 2.31	14.0 ± 1.37	7.4 ± 0.28	4.4 ± 0.52	8.1 ± 0.35	5.0 ± 0.62	12.9 ± 1.26	11.1 ± 1.87	250.3	5.81

Table S8. Environmental predictor means and standard deviations. Values were isolated from the stream temperature dataset or sourced from google earth. 'C' indicates the below-dam, current temperature estimate, and the 'H' represents the above-dam historical estimate. The intext abbreviation for each predictor is provided.

Predictor	Abbr.	11°C Acclimation Group			16°C Acclimation Group			20°C Acclimation Group		
		Estimate	Lower 89% CI	Upper 89% CI	Estimate	Lower 89% CI	Upper 89% CI	Estimate	Lower 89% CI	Upper 89% CI
Latitude [G]		-0.055 [a]	-0.233	0.122	**0.491 [b]**	**0.282**	**0.702**	*0.134 [a]*	*-0.044*	*0.313*
Current Annual Mean Monthly Maximum [N]	*CAMax*	0.069 [a]	-0.124	0.260	**0.617 [b]**	**0.424**	**0.809**	**0.368 [c]**	**0.211**	**0.527**
Historical Annual Mean Monthly Maximum [N]	*HAmax*	-0.087 [a]	-0.266	0.092	**0.270 [b]**	**0.043**	**0.501**	**0.265 [b]**	**0.087**	**0.447**
Current Annual Mean Monthly Minimum [N]	*CAMin*	**0.270 [a]**	**0.078**	**0.466**	**0.593 [b]**	**0.390**	**0.800**	**0.578 [b]**	**0.373**	**0.781**
Historical Annual Mean Monthly Minimum [N]	*HAMin*	**0.310 [a]**	**0.072**	**0.546**	**0.702 [b]**	**0.530**	**0.874**	**0.508 [ab]**	**0.341**	**0.677**
Current Annual Temperature Range [N]	*CARange*	-0.009 [a]	-0.187	0.169	**0.556 [b]**	**0.344**	**0.772**	**0.274 [c]**	**0.115**	**0.435**
Historical Annual Temperature Range [N]	*HARange*	**-0.276 [ab]**	**-0.450**	**-0.101**	**-0.532 [a]**	**-0.728**	**-0.337**	**-0.179 [b]**	**-0.347**	**-0.011**
Current Rearing Season Maximum Monthly Average [N,P]	*CRMax*	0.118 [a]	-0.086	0.323	**0.628 [b]**	**0.449**	**0.808**	**0.334 [a]**	**0.186**	**0.483**
Historical Rearing Season Maximum Monthly Average [N,P]	*HRMax*	0.116 [a]	-0.093	0.325	**0.660 [b]**	**0.486**	**0.830**	**0.356 [a]**	**0.208**	**0.501**
Current Rearing Core Maximum Monthly Average [N,P]	*CRCMax*	**0.249 [a]**	**0.058**	**0.438**	**0.528 [b]**	**0.328**	**0.729**	**0.622 [b]**	**0.420**	**0.822**
Historical Rearing Core Maximum Monthly Average [N,P]	*HRCMax*	**0.266 [a]**	**0.030**	**0.502**	**0.714 [b]**	**0.527**	**0.901**	**0.600 [b]**	**0.417**	**0.781**
Current Rearing Season Average Monthly Average [N,P]	*CRAve*	**0.304 [a]**	**0.110**	**0.498**	**0.579 [b]**	**0.385**	**0.775**	**0.580 [b]**	**0.388**	**0.769**
Historical Rearing Season Average Monthly Average [N,P]	*HRAve*	**0.307 [a]**	**0.074**	**0.540**	**0.719 [b]**	**0.536**	**0.900**	**0.533 [ab]**	**0.362**	**0.705**
Migration Distance [R]	*Mig.D*	*-0.122 [a]*	*-0.295*	*0.052*	0.058 [ab]	-0.181	0.296	*0.135 [b]*	*-0.043*	*0.314*
Migration Slope	*Mig.S*	*-0.138 [a]*	*-0.339*	*0.064*	**-0.653 [b]**	**-0.828**	**-0.476**	**-0.364 [a]**	**-0.526**	**-0.201**

Table S9. GLMM covariate estimates of between environmental predictors and growth rate for fall-run populations, including the Trinity Hatchery. The letter superscript denotes significant difference between acclimation groups for a given row. The upper and lower bounds of the 89% credible interval are given. Light gray text indicates no significant correlation, italics indicates weak significance (70% credible interval) and bold indicates strong significance (89% credible interval).

Predictor	Abbr.	11°C Acclimation Group			16°C Acclimation Group			20°C Acclimation Group		
		Estimate	Lower 89% CI	Upper 89% CI	Estimate	Lower 89% CI	Upper 89% CI	Estimate	Lower 89% CI	Upper 89% CI
Latitude		-0.131 ^{ab†}	-0.271	0.212	0.063 ^{a†}	-0.133	0.255	**-0.216** ^{b†}	**-0.382**	**-0.050**
Current Annual Mean Monthly Maximum	CAMax	-0.109 ^{a†}	-0.248	0.030	0.002 ^{ab†}	-0.188	0.192	0.090 ^{b†}	-0.055	0.234
Historical Annual Mean Monthly Maximum	HAmax	-0.136 ^{a†}	-0.279	0.008	0.073 ^{ab†}	-0.114	0.260	0.138 ^{b†}	-0.011	0.288
Current Annual Mean Monthly Minimum	CAMin	0.096 ^{ab†}	-0.050	0.245	-0.036 ^{a†}	-0.237	0.166	**0.268** ^{b}	**0.093**	**0.442**
Historical Annual Mean Monthly Minimum	HAMin	0.035 ^{a†}	-0.130	0.199	0.158 ^{a†}	-0.049	0.364	**0.193** ^{a}	**0.024**	**0.363**
Current Annual Temperature Range	CARange	-0.135 ^{a†}	-0.273	0.002	0.024 ^{a†}	-0.170	0.221	0.033 ^{a†}	-0.110	0.176
Historical Annual Temperature Range	HARange	-0.128 ^{a†}	-0.266	0.011	-0.008 ^{a†}	-0.201	0.187	0.038 ^{a†}	-0.102	0.176
Current Rearing Season Maximum Monthly Average	CRMax	-0.136 ^{a†}	-0.274	0.005	-0.046 ^{a†}	-0.247	0.154	0.000 ^{a†}	-0.143	0.144
Historical Rearing Season Maximum Monthly Average	HRMax	**-0.139** ^{a†}	**-0.272**	**-0.004**	0.091 ^{a†}	-0.111	0.292	0.025 ^{a†}	-0.124	0.173
Current Rearing Core Maximum Monthly Average	CRCMax	0.060 ^{a†}	-0.084	0.201	-0.069 ^{a†}	-0.255	0.119	**0.323** ^{b}	**0.143**	**0.502**
Historical Rearing Core Maximum Monthly Average	HRCMax	0.038 ^{a†}	-0.136	0.212	-0.112 ^{ab†}	-0.084	0.308	**0.316** ^{b}	**0.138**	**0.492**
Current Rearing Season Average Monthly Average	CRAve	0.103 ^{ab†}	-0.041	0.248	-0.061 ^{a†}	-0.250	0.127	**0.285** ^{b}	**0.107**	**0.459**
Historical Rearing Season Average Monthly Average	HRAve	0.066 ^{a†}	-0.100	0.233	0.140 ^{a†}	-0.063	0.341	**0.232** ^{a}	**0.060**	**0.404**
Migration Distance	Mig.D	-0.119 ^{a}	-0.262	0.022	-0.031 ^{ab}	-0.162	0.223	0.107 ^{b}	-0.042	0.254
Migration Slope	Mig.S	0.063 ^{a†}	-0.079	0.205	0.008 ^{a†}	-0.219	0.232	-0.026 ^{a†}	-0.172	0.124

Table S10: GLMM covariate estimates of between environmental predictors and growth rate for fall-run populations, excluding the Trinity Hatchery. The letter superscript denotes significance (89% credible interval) between acclimation groups for a given row. The upper and lower 89% credible interval are given. Light gray text indicates no significant interaction, italics indicates weak significance (70% credible interval) and bold indicates strong significance (89% credible interval). † indicates the trend of this result differs when the Trinity hatchery growth data is included. See supplemental data for environmental associations including the Trinity population.

Environmental Predictor	Abbr.	11°C Acclimation Group			16°C Acclimation Group			20°C Acclimation Group		
		Estimate	Lower 89% CI	Upper 89% CI	Estimate	Lower 89% CI	Upper 89% CI	Estimate	Lower 89% CI	Upper 89% CI
Latitude		-0.026 [a]	-0.292	0.237	*-0.155 [a]*	*-0.417*	*0.107*	*0.255 [b]*	*-0.013*	*0.518*
Current Annual Mean Monthly Maximum	*CAMax*	-0.087 [a]	-0.359	0.201	*0.250 [b]*	*-0.033*	*0.547*	*0.188 [b]*	*-0.092*	*0.476*
Historical Annual Mean Monthly Maximum	*HAmax*	-0.024 [a]	-0.262	0.210	**0.271 [b]**	**0.031**	**0.498**	*0.211 [b]*	*-0.019*	*0.435*
Current Annual Mean Monthly Minimum	*CAMin*	-0.124 [a]	-0.394	0.160	*0.236 [b]*	*-0.037*	*0.521*	0.042 [c]	-0.230	0.331
Historical Annual Mean Monthly Minimum	*HAMin*	-0.130 [a]	-0.376	0.122	*0.155 [b]*	*-0.096*	*0.412*	**0.345 [c]**	**0.097**	**0.600**
Current Annual Temperature Range	*CARange*	-0.063 [a]	-0.330	0.211	0.142 [b]	-0.137	0.431	0.156 [b]	-0.113	0.435
Historical Annual Temperature Range	*HARange*	0.122 [a]	-0.175	0.432	0.098 [a]	-0.206	0.404	-0.122 [b]	-0.422	0.181
Current Rearing Season Maximum Monthly Average	*CRMax*	-0.137 [a]	-0.427	0.164	0.101 [b]	-0.200	0.414	0.143 [b]	-0.153	0.451
Historical Rearing Season Maximum Monthly Average	*HRMax*	-0.128 [a]	-0.401	0.160	*0.089 [b]*	*-0.191*	*0.384*	**0.287 [c]**	**0.014**	**0.575**
Current Rearing Core Maximum Monthly Average	*CRCMax*	-0.135 [a]	-0.413	0.146	*0.232 [b]*	*-0.045*	*0.514*	-0.047 [a]	-0.330	0.245
Historical Rearing Core Maximum Monthly Average	*HRCMax*	-0.114 [a]	-0.354	0.128	*0.211 [b]*	*-0.030*	*0.457*	**0.273 [b]**	**0.031**	**0.519**
Current Rearing Season Average Monthly Average	*CRAve*	-0.151 [a]	-0.444	0.151	*0.201 [b]*	*-0.093*	*0.505*	-0.027 [a]	-0.325	0.281
Historical Rearing Season Average Monthly Average	*HRAve*	-0.117 [a]	-0.365	0.131	*0.162 [b]*	*-0.092*	*0.412*	**0.302 [b]**	**0.052**	**0.555**
Migration Distance	*Mig.D*	-0.002 [a]	-0.261	0.255	**0.265 [b]**	**0.008**	**0.518**	0.133 [ab]	-0.116	0.381
Migration Slope	*Mig.S*	*0.198 [a]*	*-0.113*	*0.502*	-0.154 [b]	-0.478	0.157	*-0.239 [b]*	*-0.557*	*0.068*

Table S11: GLMM covariate estimates of fifteen environmental predictor variables and CT_{max} for fish at three acclimation temperatures. Estimates reflect Z-score standardized model parameters. Estimate is the mean associations between a given environmental predictor and CT_{max}. The superscript denotes significance (89% credible interval) between acclimation groups for a given row. The upper and lower 89% credible interval are given. Light gray text indicates no significant interaction, italics indicates weak significance (70% credible interval) and bold indicates strong significance (89% credible interval).

		11 °C Acclimation Temperature			16 °C Acclimation Temperature			20 °C Acclimation Temperature		
	Test Temperature	11°C	16°C	20°C	11°C	16°C	20°C	11°C	16°C	20°C
Environmental Predictor	Latitude	0.019 ± 0.107	0.026 ± 0.105	0.032 ± 0.106	0.036 ± 0.107	0.036 ± 0.105	0.035 ± 0.106	-0.031 ± 0.107	0.004 ± 0.106	0.032 ± 0.106
	CAMax	**-0.145 ± 0.099**	*-0.085 ± 0.097*	-0.038 ± 0.099	-0.019 ± 0.100	-0.038 ± 0.098	-0.054 ± 0.099	-0.063 ± 0.100	-0.070 ± 0.097	-0.076 ± 0.098
	HAmax	**-0.213 ± 0.058**	**-0.166 ± 0.055**	**-0.128 ± 0.057**	-0.035 ± 0.057	*-0.063 ± 0.054*	**-0.086 ± 0.055**	**-0.129 ± 0.057**	**-0.128 ± 0.054**	**-0.128 ± 0.054**
	CAMin	-0.034 ± 0.112	-0.015 ± 0.110	0.001 ± 0.111	-0.058 ± 0.112	-0.050 ± 0.110	-0.043 ± 0.111	0.034 ± 0.112	0.013 ± 0.111	-0.003 ± 0.111
	HAMin	0.014 ± 0.109	0.015 ± 0.106	0.016 ± 0.107	-0.070 ± 0.108	-0.043 ± 0.106	-0.021 ± 0.107	0.020 ± 0.108	0.019 ± 0.106	0.019 ± 0.107
	CARange	*-0.139 ± 0.095*	*-0.085 ± 0.093*	-0.041 ± 0.095	0.002 ± 0.095	-0.021 ± 0.093	-0.040 ± 0.094	*-0.083 ± 0.095*	*-0.081 ± 0.093*	*-0.080 ± 0.093*
	HARange	**-0.169 ± 0.090**	**-0.134 ± 0.087**	*-0.106 ± 0.088*	0.032 ± 0.089	-0.012 ± 0.087	-0.047 ± 0.088	*-0.114 ± 0.089*	*-0.111 ± 0.087*	*-0.110 ± 0.087*
	CRMax	-0.065 ± 0.113	-0.032 ± 0.112	-0.005 ± 0.113	-0.056 ± 0.113	-0.054 ± 0.112	-0.053 ± 0.112	0.016 ± 0.114	-0.017 ± 0.112	-0.043 ± 0.113
	HRMax	-0.069 ± 0.110	-0.014 ± 0.108	0.030 ± 0.109	0.002 ± 0.109	-0.007 ± 0.108	-0.014 ± 0.109	-0.026 ± 0.109	-0.029 ± 0.108	-0.032 ± 0.108
	CRCMax	-0.062 ± 0.105	-0.020 ± 0.103	0.014 ± 0.104	-0.018 ± 0.105	-0.018 ± 0.103	-0.017 ± 0.104	-0.040 ± 0.104	-0.035 ± 0.102	-0.031 ± 0.103
	HRCMax	-0.024 ± 0.107	-0.015 ± 0.105	-0.008 ± 0.106	-0.077 ± 0.106	-0.056 ± 0.105	-0.039 ± 0.106	0.008 ± 0.107	-0.006 ± 0.105	-0.016 ± 0.106
	CRAve	-0.032 ± 0.111	-0.004 ± 0.109	0.018 ± 0.110	-0.061 ± 0.111	-0.050 ± 0.109	-0.041 ± 0.109	0.033 ± 0.111	-0.002 ± 0.110	-0.030 ± 0.111
	HRAve	0.009 ± 0.113	0.010 ± 0.111	0.010 ± 0.111	-0.076 ± 0.111	-0.048 ± 0.110	-0.026 ± 0.111	0.017 ± 0.112	0.007 ± 0.111	-0.002 ± 0.111
	Mig.D	**-0.219 ± 0.063**	**-0.170 ± 0.060**	**-0.131 ± 0.061**	-0.016 ± 0.063	*-0.054 ± 0.060*	*-0.084 ± 0.061*	**-0.129 ± 0.063**	**-0.125 ± 0.059**	**-0.122 ± 0.060**
	Mig.S	0.022 ± 0.110	-0.018 ± 0.108	-0.051 ± 0.110	0.032 ± 0.110	0.023 ± 0.108	0.015 ± 0.109	-0.017 ± 0.110	-0.021 ± 0.108	-0.024 ± 0.109

Table S12: GLMM covariate estimates of routine metabolic rate (RMR) with 15 environmental predictors. The association of each environmental predictor was assessed at three test temperatures per acclimation group. Gray text indicates no significant association. Italic text indicates a weakly significant association while bold text indicates a strongly significant association.

		11 °C Acclimation Temperature			16 °C Acclimation Temperature			20 °C Acclimation Temperature		
	Test Temperature	11°C	16°C	20°C	11°C	16°C	20°C	11°C	16°C	20°C
Environmental Predictors	Latitude	-0.098 ± 0.190	-0.119 ± 0.190	-0.132 ± 0.193	-0.081 ± 0.192	-0.130 ± 0.190	-0.159 ± 0.192	0.029 ± 0.193	0.045 ± 0.190	0.055 ± 0.192
	CAMax	*0.183 ± 0.165*	**0.293 ± 0.163**	**0.359 ± 0.166**	*0.169 ± 0.166*	**0.286 ± 0.163**	**0.355 ± 0.165**	0.115 ± 0.166	*0.160 ± 0.162*	*0.187 ± 0.163*
	HAmax	*0.198 ± 0.201*	*0.258 ± 0.200*	*0.293 ± 0.203*	0.105 ± 0.202	*0.185 ± 0.200*	*0.232 ± 0.202*	0.071 ± 0.203	0.104 ± 0.199	0.124 ± 0.201
	CAMin	0.072 ± 0.199	0.133 ± 0.198	0.169 ± 0.201	0.115 ± 0.201	*0.209 ± 0.198*	*0.265 ± 0.200*	0.112 ± 0.201	0.144 ± 0.199	0.163 ± 0.201
	HAMin	-0.081 ± 0.232	-0.100 ± 0.233	-0.111 ± 0.236	-0.034 ± 0.235	0.009 ± 0.232	0.034 ± 0.233	0.124 ± 0.234	0.158 ± 0.232	0.178 ± 0.233
	CARange	*0.171 ± 0.187*	*0.263 ± 0.186*	**0.317 ± 0.189**	0.146 ± 0.188	*0.234 ± 0.186*	**0.286 ± 0.189**	0.085 ± 0.189	0.120 ± 0.185	0.141 ± 0.186
	HARange	*0.213 ± 0.225*	*0.274 ± 0.225*	*0.310 ± 0.228*	0.117 ± 0.227	0.148 ± 0.225	0.167 ± 0.226	-0.029 ± 0.228	-0.023 ± 0.224	-0.019 ± 0.224
	CRMax	0.122 ± 0.183	*0.226 ± 0.182*	**0.288 ± 0.185**	0.155 ± 0.185	*0.251 ± 0.182*	**0.308 ± 0.184**	0.117 ± 0.185	0.153 ± 0.181	*0.175 ± 0.182*
	HRMax	0.059 ± 0.199	0.113 ± 0.199	0.146 ± 0.202	0.072 ± 0.201	0.145 ± 0.199	*0.188 ± 0.200*	0.127 ± 0.202	0.163 ± 0.198	*0.184 ± 0.200*
	CRCMax	*0.137 ± 0.147*	**0.233 ± 0.145**	**0.290 ± 0.148**	*0.156 ± 0.149*	**0.288 ± 0.146**	**0.367 ± 0.148**	0.092 ± 0.150	*0.113 ± 0.147*	*0.126 ± 0.150*
	HRCMax	0.004 ± 0.221	0.007 ± 0.221	0.009 ± 0.224	0.026 ± 0.222	0.093 ± 0.220	0.132 ± 0.221	0.138 ± 0.223	0.167 ± 0.220	0.185 ± 0.222
	CRAve	*0.093 ± 0.132*	*0.152 ± 0.130*	*0.187 ± 0.134*	*0.133 ± 0.134*	**0.213 ± 0.130**	**0.260 ± 0.132**	0.093 ± 0.135	*0.114 ± 0.132*	*0.126 ± 0.135*
	HRAve	-0.037 ± 0.190	-0.046 ± 0.189	-0.052 ± 0.192	-0.024 ± 0.191	0.035 ± 0.189	0.070 ± 0.190	0.175 ± 0.191	*0.167 ± 0.189*	*0.163 ± 0.191*
	Mig.D	*0.209 ± 0.208*	*0.282 ± 0.207*	**0.326 ± 0.209**	0.120 ± 0.209	*0.194 ± 0.207*	*0.239 ± 0.209*	0.047 ± 0.208	0.083 ± 0.205	0.105 ± 0.206
	Mig.S	-0.020 ± 0.201	-0.095 ± 0.201	-0.139 ± 0.204	-0.095 ± 0.204	*-0.177 ± 0.201*	*-0.226 ± 0.202*	-0.145 ± 0.203	*-0.200 ± 0.200*	*-0.232 ± 0.202*

Table S13: GLMM covariate estimates of maximum metabolic rate (MMR) with 15 environmental predictors. The association of each environmental predictor was assessed at three test temperatures per acclimation group. Gray text indicates no significant association. Italic text indicates a weakly significant association while bold text indicates a strongly significant association.

		11 °C Acclimation Temperature			16 °C Acclimation Temperature			20 °C Acclimation Temperature		
	Test Temperature	11°C	16°C	20°C	11°C	16°C	20°C	11°C	16°C	20°C
Environmental Predictors	Latitude	-0.107 ± 0.230	-0.155 ± 0.230	-0.168 ± 0.228	-0.094 ± 0.230	-0.152 ± 0.231	*-0.210 ± 0.229*	0.073 ± 0.231	0.065 ± 0.230	0.069 ± 0.229
	CAMax	0.148 ± 0.169	**0.312 ± 0.168**	**0.391 ± 0.166**	0.076 ± 0.169	*0.230 ± 0.169*	**0.337 ± 0.167**	0.052 ± 0.169	*0.151 ± 0.169*	*0.194 ± 0.167*
	HAmax	*0.212 ± 0.210*	*0.293 ± 0.208*	**0.336 ± 0.207**	-0.010 ± 0.211	0.096 ± 0.211	0.183 ± 0.209	-0.013 ± 0.211	0.076 ± 0.209	0.121 ± 0.208
	CAMin	0.025 ± 0.214	0.123 ± 0.213	0.162 ± 0.211	0.088 ± 0.212	*0.213 ± 0.213*	*0.305 ± 0.212*	0.069 ± 0.213	0.134 ± 0.212	0.150 ± 0.211
	HAMin	-0.108 ± 0.237	-0.160 ± 0.235	-0.182 ± 0.233	-0.028 ± 0.235	0.038 ± 0.235	0.066 ± 0.234	0.168 ± 0.235	0.204 ± 0.234	*0.212 ± 0.233*
	CARange	0.143 ± 0.204	*0.275 ± 0.203*	**0.340 ± 0.201**	0.044 ± 0.204	0.148 ± 0.204	*0.228 ± 0.202*	0.019 ± 0.205	0.098 ± 0.204	0.137 ± 0.202
	HARange	*0.245 ± 0.229*	*0.336 ± 0.227*	**0.388 ± 0.226**	0.016 ± 0.229	0.053 ± 0.229	0.100 ± 0.228	-0.146 ± 0.229	-0.108 ± 0.229	-0.071 ± 0.228
	CRMax	0.062 ± 0.200	*0.216 ± 0.199*	*0.286 ± 0.197*	0.079 ± 0.200	*0.206 ± 0.200*	*0.281 ± 0.198*	0.080 ± 0.200	0.153 ± 0.200	*0.180 ± 0.199*
	HRMax	0.016 ± 0.219	0.087 ± 0.218	0.117 ± 0.216	0.004 ± 0.219	0.107 ± 0.219	0.159 ± 0.218	0.120 ± 0.219	0.184 ± 0.219	*0.207 ± 0.217*
	CRCMax	0.109 ± 0.174	*0.263 ± 0.175*	**0.323 ± 0.172**	0.135 ± 0.174	**0.312 ± 0.175**	**0.434 ± 0.173**	0.049 ± 0.173	0.123 ± 0.174	0.136 ± 0.173
	HRCMax	-0.038 ± 0.228	-0.053 ± 0.228	-0.058 ± 0.225	0.007 ± 0.225	0.113 ± 0.226	0.167 ± 0.225	0.139 ± 0.226	0.189 ± 0.225	0.198 ± 0.224
	CRAve	0.065 ± 0.189	*0.196 ± 0.190*	*0.244 ± 0.187*	0.131 ± 0.188	**0.301 ± 0.189**	**0.409 ± 0.187**	0.070 ± 0.188	0.135 ± 0.188	0.140 ± 0.187
	HRAve	-0.086 ± 0.238	-0.134 ± 0.236	-0.155 ± 0.234	-0.012 ± 0.234	0.072 ± 0.235	0.108 ± 0.235	0.159 ± 0.235	0.193 ± 0.235	0.196 ± 0.234
	Mig.D	*0.238 ± 0.207*	**0.343 ± 0.206**	**0.400 ± 0.204**	0.003 ± 0.208	0.091 ± 0.208	0.179 ± 0.206	-0.050 ± 0.208	0.037 ± 0.207	0.089 ± 0.206
	Mig.S	-0.014 ± 0.215	-0.148 ± 0.215	*-0.217 ± 0.213*	-0.025 ± 0.215	-0.125 ± 0.215	*-0.201 ± 0.214*	-0.075 ± 0.215	-0.170 ± 0.215	*-0.216 ± 0.214*

Table S14: GLMM covariate estimates of aerobic scope (AS) with 15 environmental predictors. The association of each environmental predictor was assessed at three test temperatures per acclimation group. Gray text indicates no significant association. Italic text indicates a weakly significant association while bold text indicates a strongly significant association.

Population	Current Reach	Current Spawning Range (km)	Historical Reaches	Historical Spawning Range (km)
Feather River	Feather River	26	West Branch Feather River, North Fork Feather River, Middle Fork Feather River, South Fork Feather River	59
Coleman	Battle Creek	9.7	Battle Creek	9.8
Trinity River	Trinity River	56	Stuart Fork, Coffee Creek, East Fork Trinity River, Trinity River (Upper)	52.99
Elk River	Elk River	12.3	Elk River (upper)	13.1
Trask River	Trask River	16	Trask River (upper)	14
Priest Rapids	Columbia River	92	Columbia River (upper)	93

Table S15: Geographic data for extracting stream temperature data for each population. Current spawning range is the length of the 'Current Reach' downstream of the hatchery that was isolated for identifying temperature data. Historical Reaches are the named rivers for which historical temperature data was isolated above the hatchery and adjacent reservoirs. Historical Spawning Range is the number of kilometers isolated for identifying pre-dam temperature data. This data is visualized in Figure 1.
